# Integrating pharmacokinetics and network analysis to investigate the mechanism of Moutan Cortex in blood-heat and blood stasis syndrome

**DOI:** 10.1186/s13020-022-00657-w

**Published:** 2022-09-14

**Authors:** Qiuli Ye, Ying Zhang, Donghui Yan, Yue Sun, Ming Li, Hui Cao, Shumei Wang, Jiang Meng

**Affiliations:** 1 School of Traditional Chinese Medicine, Guangdong Pharmaceutical University/Key Laboratory of Digital Quality Evaluation of Chinese Materia Medica, State Administration of Traditional Chinese Medicine (TCM)/Engineering Technology Research Center for Chinese Materia Medica Quality of Universities in Guangdong Province, Guangzhou, 510006 China; 2 College of Pharmacy, Jinan University, Guangzhou, 510632 China; 3 Medical Research Center, Guangdong Second Provincial General Hospital, Guangzhou, Guangdong China

**Keywords:** Raw Moutan Cortex, UHPLC-DAD, Absorbed compounds, Pharmacokinetics, Network analysis, HUVECs

## Abstract

**Background:**

Raw Moutan Cortex (RMC) has been used in China and other Asian countries for thousands of years. Its medical application is the treatment of cooling blood and promoting blood circulation. However, its therapeutic mechanism is still undefined.

**Methods:**

In this study, the pharmacokinetics strategy that integrated network analysis was employed to explore the mechanism of RMC in blood-heat and blood stasis syndrome (BHS) model rats. Firstly, Ultra-High performance Liquid Chromatography coupled with Diode Array Detector (UHPLC-DAD) method was developed to determine nine absorbed compounds in rat serum in BHS and normal rats after oral administration of RMC extract respectively. Then the pharmacology network was established based on the relationship between nine compounds absorbed into the blood and BHS targets. Finally, the predicted hub targets were validated experimentally in human umbilical vein endothelial cells (HUVECs).

**Results:**

Pharmacokinetic study showed that the pharmacokinetic parameters of nine absorbed compounds had significant differences between BHS and normal groups (*p* < 0.05). Network analysis showed that 8 target genes, namely, F2, F10, F7, PLAU, MAPK14, MAPK10, AKT1, and NOS3 may be the primary targets regulated by RMC for the treatment of BHS. Among them, targets (F2, F10, F7 and MAPK14, MAPK10, AKT) and 4 active ingredients (paeonol, paeoniflorin, quercetin and oxypaeoniflorin) were selected for evaluating the reliability in vitro experiments, which revealed that the mechanism of RMC against BHS syndrome may inhibit inflammatory pathways and regulate coagulation cascades pathway for cooling and promoting blood circulation.

**Conclusion:**

The proposed pharmacokinetics study integrated network analysis strategy provides a combination method to explore the therapeutic mechanism of RMC on BHS.

**Graphical Abstract:**

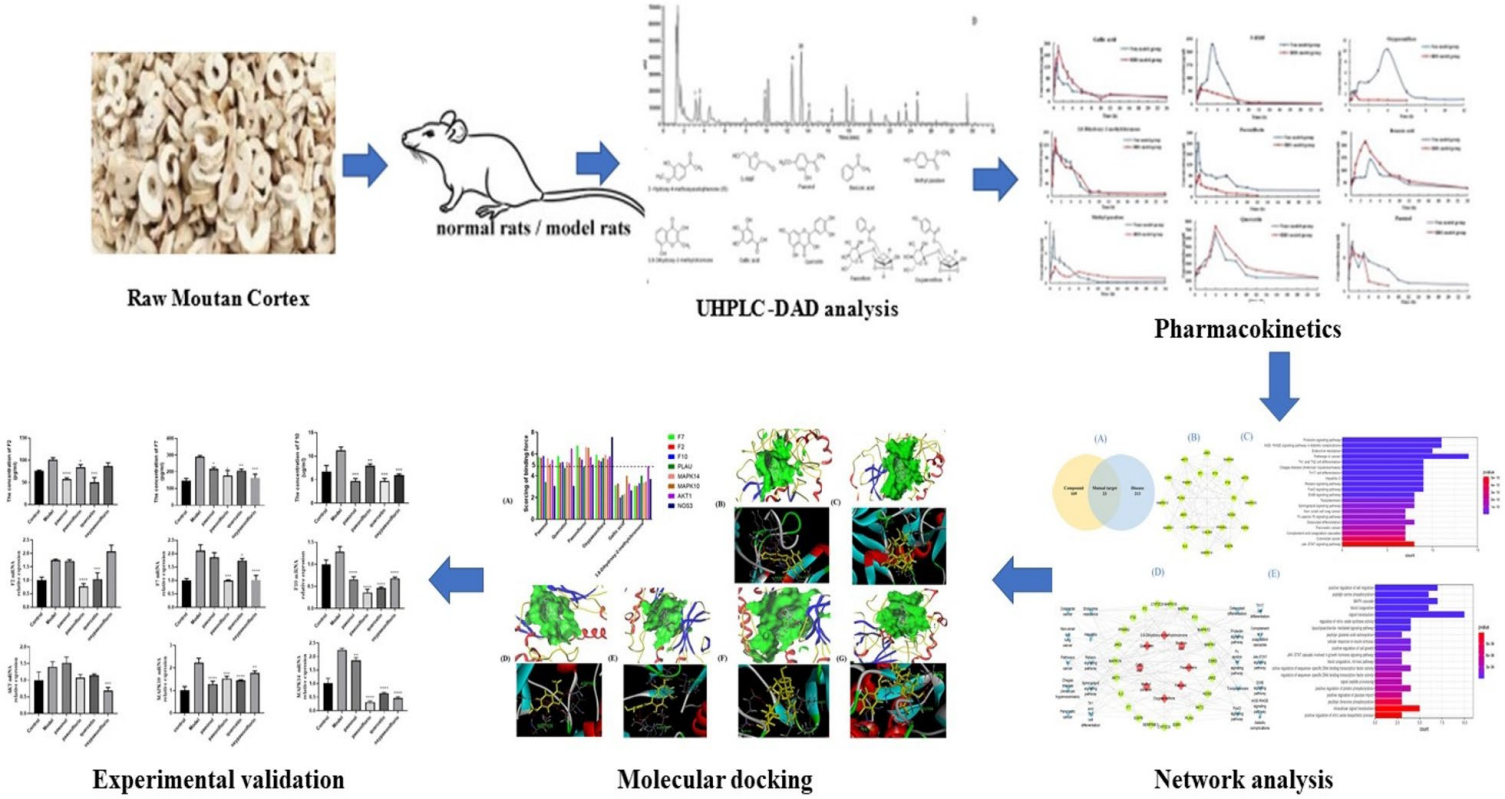

**Supplementary Information:**

The online version contains supplementary material available at 10.1186/s13020-022-00657-w.

## Background

Blood-heat and blood stasis syndrome (BHS) is a pathological condition in the blood circulation which may cause the development of various diseases, such as acute cerebral hemorrhage, stasis fever, coronary heart disease, etc. [1]. Previous studies have shown that Chinese medicine for cooling blood and promoting blood circulation has anti-platelet aggregation, improve microcirculation, regulate immune function and anti-inflammatory. Definitely, its application prospects are broad [2, 3]. However, few research has focused on the pharmacodynamic mechanism of most Chinese medicine for cooling blood and promoting blood circulation. Its active ingredients mechanism of action is still unclear and worthy of further investigation.

Raw Moutan Cortex (RMC) is the dried root bark of the Paeonia suffruticosa Andr. It is a common traditional Chinese medicine for cooling and promoting blood circulation. It serves to clear heat and cooling blood, activates blood circulation and removing stasis, which commonly used in the clinical treatment of clearing excessive heat (typically manifesting in inflammation and related symptoms), amenorrhea and dysmenorrhea, traumatic injuries, carbuncles, ulcers and abscesses [4]. Modern pharmacological studies have shown that RMC extract could significantly inhibit the platelet aggregation rate induced by ADP, collagen and epinephrine. It also reduces the production of TXB2, inhibit plateledt aggregation induced by collagen, adenosine diphosphate and arachidonic acid, significantly prolong TT and platelet aggregation time, and reduce platelet adhesion rate [4–9]. Besides, it was reported to exhibit a variety of biological activities, including anti-inflammatory [10–12], anti-oxidative effects [13, 14], anti-diabetes [15] and protective of myocardial ischemia [16].

The main chemical components of RMC are monoterpenes and their glycosides (such as paeoniflorin, oxypaeoniflorin, etc.), phenols and their glycosides (such as paeonol, etc.), flavonoids (such as quercetin), and et al. [17]. Its main components have been proven to have good anticoagulant, anti-inflammatory, improve microcirculation, and regulate immune functions [9, 18–20]. For example, quercetin, paeonol, paeoniflorin, oxypaeoniflorin and gallic acid have the effects of inhibiting platelet aggregation, anti-inflammation and anti-oxidation [8, 21–24]. In addition, 3,8-Dihydroxy-2-methylchromone can significantly extend APTT and promote blood circulation [25]. To date, most of the research on RMC have focused on the vitro studies, such as the chemical constituents and quantitative analyses, as well as quality control protocols [26–28]. However, there are few in-depth studies of the active components of RMC in vivo. In addition, these components absorbed into the BHS after oral administration in rats is still unknown. At present, only some monomer compounds (such as paeoniflorin and paeonol) have been reported in vivo [29, 30]. Further, traditional Chinese medicine mostly works in the mode of action of “multi-component, multi-target”. The single component in vivo exposure value does not reflect the overall difference in the composition of Chinese medicine [31]. It is noteworthy that the active compounds, potential targets and pathways involved in these effects have not been systematically investigated.

From the perspective of pharmacokinetics, most traditional Chinese medicines are taken orally. When the traditional Chinese medicine ingredients are successfully absorbed into the blood and maintain a considerable concentration in the target organ, it creates an opportunity to exert their efficacy, which can provide a powerful evidence for determining the active compounds absorbed into the blood and deciphering their process in vivo [32, 33]. Network analysis is grounded in a nexus of interactions between drugs, targets and diseases. It analyzes the relationships between drugs, targets and diseases. This method that may lead to unrealistic results ignores whether the ingredients can be absorbed into the blood and its metabolism to play a curative effect [34, 35]. Therefore, in order to solve this problem, this paper proposes a pharmacokinetics and network analysis research strategy based on in vivo absorbed exposure. It lays the foundation for the search for potential active ingredients and provides a basis for revealing the mechanism of action of RMC in vivo. It also guides the clinical application of traditional Chinese medicine for cooling blood and provides a theoretical basis for activating blood under pathological conditions.

**Fig. 1:**
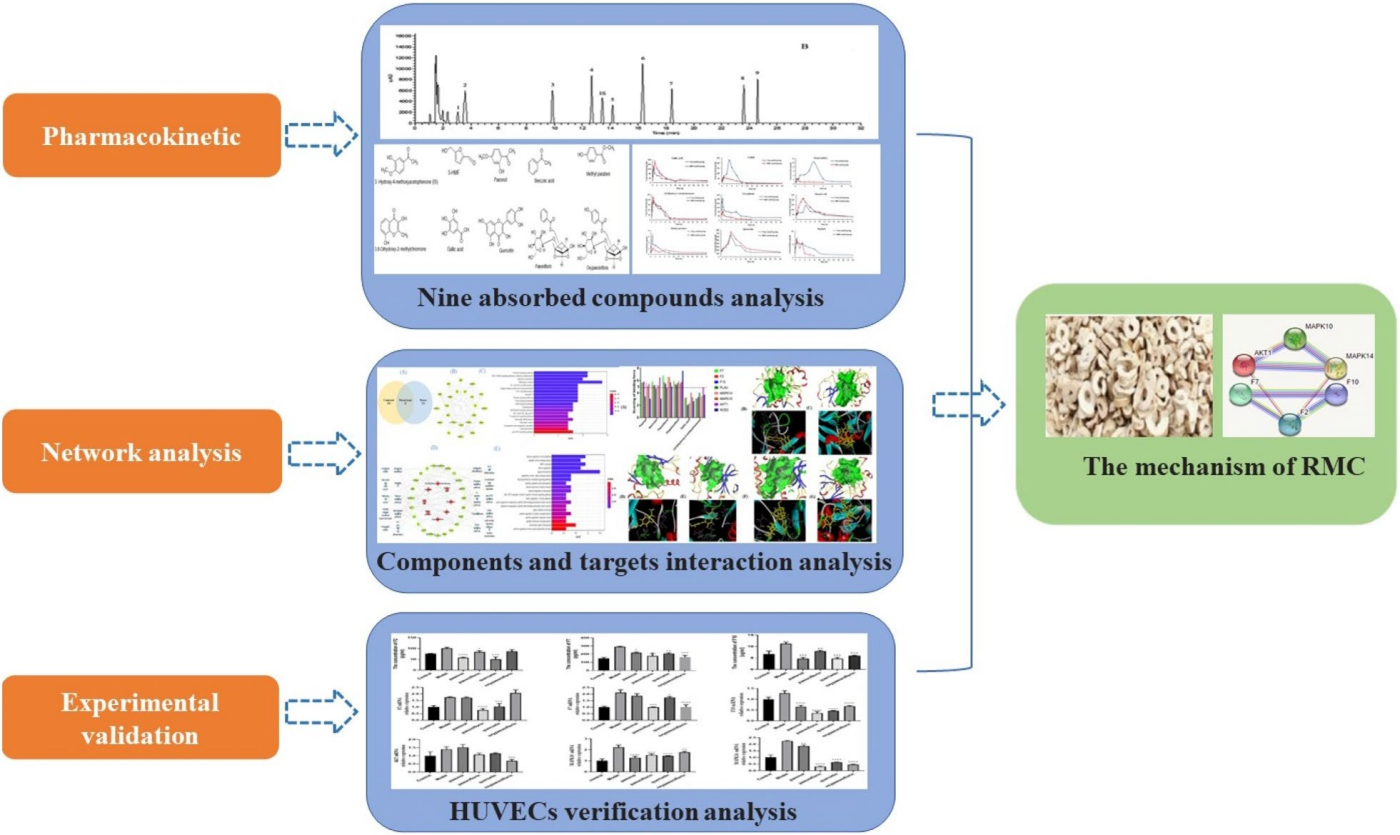
Flow chart of integrating pharmacokinetics and network analysis to investigate the mechanism of Moutan Cortex in Blood-Heat and blood Stasis Syndrome

In this study, a high-efficiency and sensitive UHPLC-DAD method was used to determine the blood concentration of nine absorbed components in the serum of RMC in normal and BHS rats at different time point. The time-concentration curve was established and the relevant main pharmacokinetic parameters was obtained. Then, the pharmacology network was established based on the relationship between nine compounds absorbed into the blood and BHS targets. Finally, hub targets in the component–target network were experimentally validated in the human umbilical vein endothelial cells (HUVECs). Pharmacokinetic-based UHPLC-DAD combined with network analysis methods can provide a reliable and appropriate method for screening the potential active components of RMC for promoting blood circulation and removing blood stasis and elucidating its mechanism of action. The detailed process is shown in Fig. 1.

## Methods

### Chemicals and reagents

**Fig. 2:**
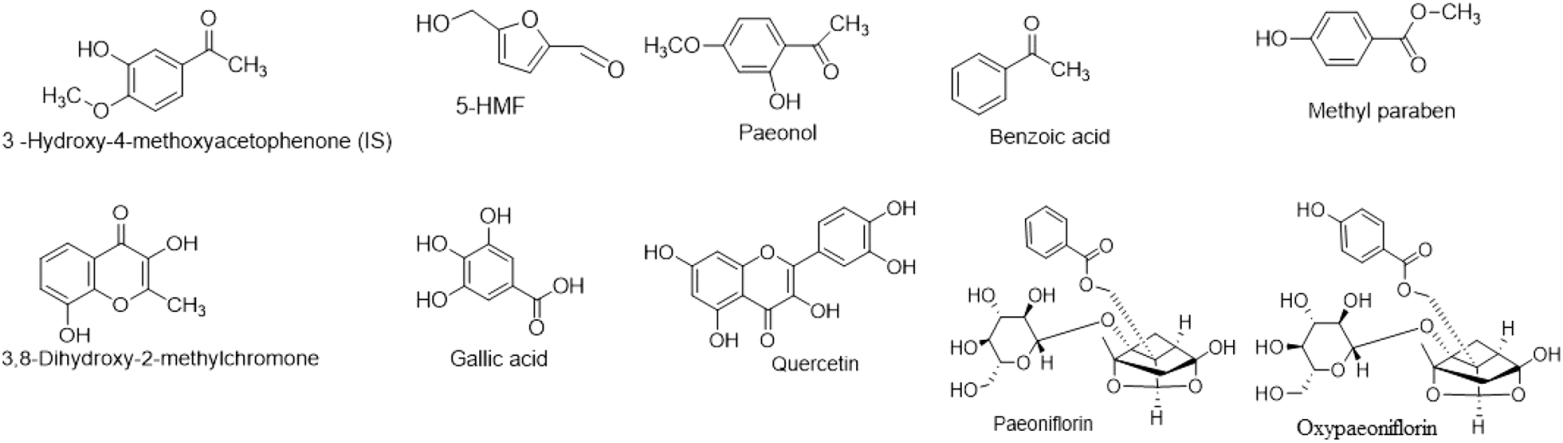
Chemical structure of the analytes and IS

Raw Moutan Cortex pieces were bought from Guangzhou Zhixin Chinese Sliced Herbal Medicine Co. Ltd. (Guangzhou, China) and identified by associate professor Jizhu Liu (School of Traditional Chinese Medicine, Guangdong Pharmaceutical University). Voucher specimens were deposited at the Herbarium Centre, Guangdong Pharmaceutical University. The reference substances, including gallic acid, 5-hydroxymethylfurfural (5-HMF), oxypaeoniflorin, paeoniflorin, benzoic acid, methyl paraben, quercetin, paeonol, 3,8-dihydroxy-2-methylchromone, 3-hydroxy-4-methoxyacetophenone (IS) with purity ≥ 98%, were obtained from Chroma Biotechnology Co. Ltd. (Chengdu, China). Their chemical structures are shown in Fig. 2. Methanol and acetonitrile were HPLC grade (Oceanpak). Purified water was Watsons water. All other reagents used in this study were analytical grade. Nitrogen was purchased from Guangzhou Junduo Gas Company (purity > 99.9%) (Guangzhou, China). ELSA kit: thromboxane B2 (A002774), 6-keto-prostaglandin F1α (A0021984), Human coagulation factor II (FII) (A011227), Human coagulation factor VIII (FVII) (A08571), Human coagulation factor X (FX) (A036278), all purchased from Shanghai Fusheng Industrial. RNAex Pro Reagent (AG11728, Accurate Biotechnology, Hunan), EvoM-MLV RT Kit with gDNA Clean for qPCR (AG11728, Accurate Biotechnology, Hunan). APTT, TT and PT Reagents (Wuhan Zhongtai Biotechnology Co., Ltd.), SC40 semi-automatic coagulation analyzer (Taizhou Zhongqin Shidi Biotechnology Co., Ltd.)

### Preparation of Moutan Cortex extract

The RMC pieces were soaked with 95% v/v ethanol at a solid–liquid ratio of 1:8 for 2 h, then reflux extracted for 2 h. After filtering, the residue was extracted again under the same conditions. The twice filtrates were collected and combined, and vacuum concentrated into an alcohol-free extractum. Last, the extractum was dissolved in water (equal to 6 g/mL of RMC) and stored in seal at 4 °C [36–38].

### Pharmacokinetic study

Twenty-four male Sprague–Dawley rats (weighing 300 ± 20 g) were purchased from Animal Experiment Center, Guangdong Academy of Medical Sciences (Certificate no. SCXK2013-0002). The rats were kept in an environmentally controlled breeding room at a temperature of 22 ± 2 °C and a relative humidity of 55 ± 10% for 1 week before starting the experiments. They were fed with standard laboratory food and water. Both the animal’s care and the study protocol were conducted according to the National Institutes of Health Guide for the Care and Use of Laboratory Animals, and followed the guidelines of the Ethics Committee of Guangdong Pharmaceutical University.

The rats were randomly divided into following four groups: normal groups (Normal groups), BHS model groups (BHS groups), normal group administrated with RMC extraction (N-RMC group), BHS group administrated with RMC extraction (BHS-RMC group), respectively. N-RMC group and BHS-RMC group rats were respectively orally administrated with RMC extraction (equal to 6 g/mL of RMC) for continuous 7 days at a dose of 10 mL/kg, once a day, 1 hour after the sixth day of administration. The rats in BHS groups and BHS-RMC group were subcutaneously injected with 10% aqueous suspension of yeast (10 mL/kg) and then subcutaneously injected with 0.1 mg/mL adrenaline hydrochloride solution twice (at a dose of 0.8 mL/kg, 4 h apart) while body temperature remained relatively steady high. Those rats were soaked in ice-water for 5 min keeping their heads outside surface between two subcutaneous injections of adrenaline hydrochloride solution [39, 40]. At the same time, the normal groups and N-RMC group rats were injected with the same dose of saline (10 mL/kg) subcutaneously. All rats were fasted for 12 h but free for water before taking the blood. On the seventh day, 0.3 mL blood samples were collected from fossa orbitalis vein at the time point of 0.083, 0.25, 0.5, 0.75, 1, 2, 3, 4, 6, 8, 10, 12 and 24 h after oral administration. All blood samples were immediately centrifuged at 13,680 (× g) for 10 min at 4 °C and the serum samples were obtained and stored at −80 °C until analysis.

### Preparation of serum sample

Firstly, 100 μL serum was spiked with 10 μL IS working solution to mix evenly, then 1 mL methanol was added to protein precipitation, and centrifuged at 1520 (× *g*) for 10 min at 4 °C. Secondly, the supernatant was collected with a clean tube carefully and filtered through 0.22 μm microporous filter head. Thirdly, the filtrate was evaporated to dryness under a stream of nitrogen at 40 °C. Finally, the residue was dissolved in 100 μL methanol and 10 μL of aliquot was injected into UHPLC-DAD system for analysis.

### Instrument and chromatographic conditions

The chromatography experiment was carried out using UPLC system (Thermo Fisher Scientific, USA). Chromatographic conditions were according to the paper published by our research group [37]. Column: Acquity UPLC^®^ BEH Shield RP_18_ Column (2.1 mm × 100 mm, 1.7 μm, Waters, USA). The column temperature was maintained at 30 °C. Mobile phase: Acetonitrile (A) and 0.1% (v/v) formic acid in water (B). The gradient conditions: 0–10 min, 5–15% A; 10–20 min, 15–35% A; 20–30 min, 35–95% A; 30–32 min, 95% A. The samples were kept at 4 °C in the auto-sampler. Flow rate and sample size, 0.2 mL/min and 2 μL; column temperature and detection wavelength, 30 °C and 254 nm.

### Preparation of reference compounds and quality control solutions

Primary stock solution of reference compounds was prepared in methanol separately at concentrations of 2.075 mg/mL (gallic acid), 3.690 mg/mL (5-HMF), 0.640 mg/mL (oxypaeoniflorin), 1.125 mg/mL (3,8-dihydroxy-2-methylchromone), 2.235 mg/mL (paeoniflorin), 3.250 mg/mL (benzoic acid), 0.260 mg/mL (Methyl paraben), 2.385 mg/mL (quercetin), 0.482 mg/mL (paeonol) and 0.460 μg/mL (3-hydroxy-4-methoxyacetophenone, IS), respectively. Working standard solutions were prepared daily. All standard stock and working solutions were stored at 4 °C.

Quality control (QC) samples were prepared by the same procedure to attain low, medium and high concentration levels. The concentrations were 186.75, 93.38 and 20.75 μg/mL for gallic acid; 405.90, 110.70 and 18.45 μg/mL for 5-HMF; 6.40, 3.20 and 0.96 μg/mL for oxypaeoniflorin; 129.38, 73.13 and 16.88 μg/mL for 3,8-dihydroxy-2-methylchromone; 145.28, 67.05 and 16.76 μg/mL for paeoniflorin; 243.75, 97.50 and 40.63 μg/mL for benzoic acid; 6.50, 3.90 and 1.30 μg/mL for methyl paraben; 715.50, 357.75 and 107.33 μg/mL for quercetin; 12.05, 7.23 and 2.41 μg/mL for paeonol.

### Method validation

The selectivity of the method was evaluated by comparing the chromatograms of blank serum, blank serum spiked with the nine analytes and IS, serum samples of N-RMC and BHS-RMC rats administrated with RMC. Calibration reference compounds of the mixture of nine components were prepared by spiking the standard mixture working solutions into 100 μL blank serum and 100 μL IS. The linearity of calibration curve was constructed by plotting the peak area ratios versus the concentration of nine components. The QC serum samples at three concentration levels were respectively analyzed in five replicates on the same day and on three consecutive validation days to evaluate the intra-day and inter-day precision and accuracy. The accuracy was expressed by the percentage difference between amount spiked and determined while the precision was presented with the relative standard deviation (RSD%). The stability of nine components was evaluated to determine the changes in three different concentrations at low, medium and high levels of QC samples subjected to three different storage conditions. Short-term stability was evaluated by assessing QC samples at the room temperature (25 °C) for 24 h. The freeze–thaw stability was inspected by implementing three freeze (− 80 °C)—thaw (room temperature, 25 °C) cycles on consecutive days. Long-term stability was determined by storing at − 80 °C for 30 days. Extraction recovery was determined by calculating the peak area ratio of QC samples to corresponding post‐extracted samples spiked with the analytes and internal standard at three concentrations of low, medium and high levels. The matrix effect was evaluated by comparison of the peak areas of nine components/IS in the post‐extracted spiked samples and standard working solutions at the same concentration. The QC serum samples at three concentration levels were analyzed in five replicates, respectively.

### The index of TXB2 and 6-Keto-PGF1 detect

It takes appropriate amount of serum and the levels of TXB2 and 6-Keto-PGFlα in the serum of rats in Normal group, BHS groups and BHS-RMC groups were detected by ELISA kit.

### Statistical analysis

The concentrations of nine components of all samples were processed by PKSolver pharmacokinetics software. Statistical comparisons among groups were performed with GraphPad Prism 8 using a One-Way ANOVA analysis. Data expressed as mean ± SD and a *p* value ≤ 0.05 was considered to be statistically significant.

### Network analysis

Targets for nine compounds: the targets of nine compounds were predicted by PharmMapper (http://lilab.ecust.edu.cn/pharmmapper/) limited to “Homo sapiens”; also including targets predicted from TCMSP (http://lsp.nwu.edu.cn/tcmspsearch.php). Subsequently, top 300 targets were predicted from the PharmMapper. Among the targets, those with z′-score > 0 was selected as the potential targets for the correlative ingredients. The PharmMapper online tool is a web server for potential drug target identification by reversed pharmacophore matching the query compound against anin-house pharmacophore model database [41].

Disease-related targets: by reviewing the literature on target proteins of traditional Chinese medicine for activating blood and cooling blood, it combined with RMC medicinal properties (bitter, acrid, slightly cold, homeward liver, kidney meridian), screen anti-inflammatory, improve hemodynamics, anti-coagulation, anti-platelet aggregation from TTD database equal to disease-related target protein. Finally, it locked the target proteins that may be related to activating blood and cooling blood [42–50].

### PPI analysis

Screen for common targets at the crossover of compounds and diseases and import into the STRING database (https://string-db.org/, version 11.0), selecting “Homo sapiens” as the species source. The protein–protein interactions (PPIs) of proteins common to compounds and diseases targets were analyzed by String at the high confidence. The purpose of PPI was to get relevant targets, and to remove targets with low confidence.

### Gene pathway analysis

Based on previous steps, compound-related targets and disease targets were prepared, and the drug-disease crossover genes were screened. Then, the Database for Annotation, Visualization and Integrated Discovery (DAVID, https://david.ncifcrf.gov/home.jsp, ver. 6.8) was used to identify the signal pathways and biological functions related to compound-disease target genes, which were analyzed by KEGG pathway enrichment (*p* < 0.05) and GO enrichment (*p* < 0.05).

### Network construction

The compounds-target (C-T) and compounds-target -pathway network was built by network analysis software (Cytoscape 3.3.0).

### Molecular docking

The molecular structures of the compounds were downloaded from PUBMED.

(https://www.ncbi.nlm.nih.gov/) and then imported into Oppen Babel (2.4.1) software to transform the three-dimensional (3D) structure to mol2 file format. Several key targets are extracted from the major hub network, including F2, F7, F10, PLAU, MAPK14, MAPK10, AKT1 and NOS3. The 3D structures of Prothrombin (F2, pdb = 3tu7), Coagulation factor VII (F7, pdb = 4zxy), Coagulation factor FX (F10, pdb = 1g2m), Urokinase-type plasminogen activator (PLAU, pdb = 1ejn), Mitogen-activated protein kinase 14 (MAPK14, pdb = 1a9u), Mitogen-activated protein kinase 10 (MAPK10, pdb = 3cgf), threonine-protein kinase(AKT1, pdb = 7nh5) and Nitric oxide synthase (NOS3, pdb = 1maj) were downloaded from the PDB database (https://www.rcsb.org/) (source: Homo sapiens; method: X-ray; resolution > 1.5; number of ligands > 1) using Sybyl-7.3 for processing. After removing the water molecules residues, hydrogenation, use the Surflex-Dock program Docking with its own ligand, the score obtained is used as the threshold value of the target. The compounds were docked with the target protein obtained by screening and scored. The target with a score greater than the target threshold (> 5) regarded as the master targets for RMC [51]. Then docking sites were analyzed using Discovery Studio 4.5 software to observe the Interaction between compound and target.

### Cell culture and treatments

TNF-α was used to induce endothelial factor damage in Human Umbilical Vein Endothelial Cells (HUVEC) leading to inflammation and coagulation dysfunction. HUVECs were maintained in ECM complete growth medium (ECM, 5% FBS, 1% P/S and 1% EGCS). All cells were cultured at 37 °C in a humidified atmosphere containing 5% CO_2_. After three or four passages, the HUVECs were digested with 0.25% trypsin and seeded in a 6-well plate with a density of 1 × 10^5^ cells/mL for ELISA analysis and RT-qPCR. First, we determined the effects of different doses of drug on the viability of HUVECs cells using the MTT methods (MTT results are shown in Additional file 1: Fig. S1).The cells used for experiment were divided into the following groups: control group (giving complete growth medium), model group (giving 25 ng/mL TNF-α), paeonol (50 μg/mL + 25 ng/mL TNF-α), paeoniflorin (500 μg/mL + 25 ng/mL TNF-α), quercetin (1 μg/mL + 25 ng/mL TNF-α) and oxypaeoniflorin (500 μg/mL + 25 ng/mL TNF-α) for 8 h, respectively. Afterwards, take the supernatant and measure according to the ELISA kit, and detect the OD value at the wavelength of 450. Meanwhile, Total RNAs were extracted according to the manufacturer’s instructions, which were used to generate cDNA using EvoM-MLV RT Kit with gDNA Clean for qPCR. ChamQTMUniversalSYBR qPCR Master Mix (Vazyme Biotech Co., Ltd, Q711-02) was performed on a Real-time PCR System. GAPDH was used as an internal control to normalize RNA expression. Primers were from Bioengineering (Shanghai Co., Ltd) and the primers information used in RT-qPCR were shown in Additional file 1: Table S1.

### Coagulation test

New Zealand white rabbits purchased from Laboratory Animal Center, Guangzhou University of Chinese Medicine (License No: SCXK (YUE) 2019–0035, Guangzhou, China). New Zealand white rabbits, after being fasted but with free access to water for 8 h, were anesthetized by intraperitoneal injection of 10.0% chloral hydrate at 0.3 mL/100 g. The blood collected from the carotid artery was anticoagulated with 3.8% sodium citrate (blood and dosage volume ratio of 9:1), and transferred to a vacuum blood-collection tube, which was then slowly inverted and mixed for 3 to 4 times, and centrifuged at 860 (× *g*) for 10 min to obtain the supernatant. Paeoniflorin, oxypaeoniflorin, quercetin and paeonol were separately dissolved in methanol to prepare the solution at a concentration of 250 μg/mL, 500 μg/mL,1 μg/mL, 50 μg/mL respectively, at the same time with the methanol solvent used as a blank control. Whereafter, 10 μL of monomer solution was added under the kit instructions to determine APTT, PT and TT using coagulation method. In this experiment, each sample was measured three times, with the results of the coagulation test presented as mean ± standard deviation ($\overline{{\text{x}}}$  ± SD).

## Results

### Method validation

**Fig. 3:**
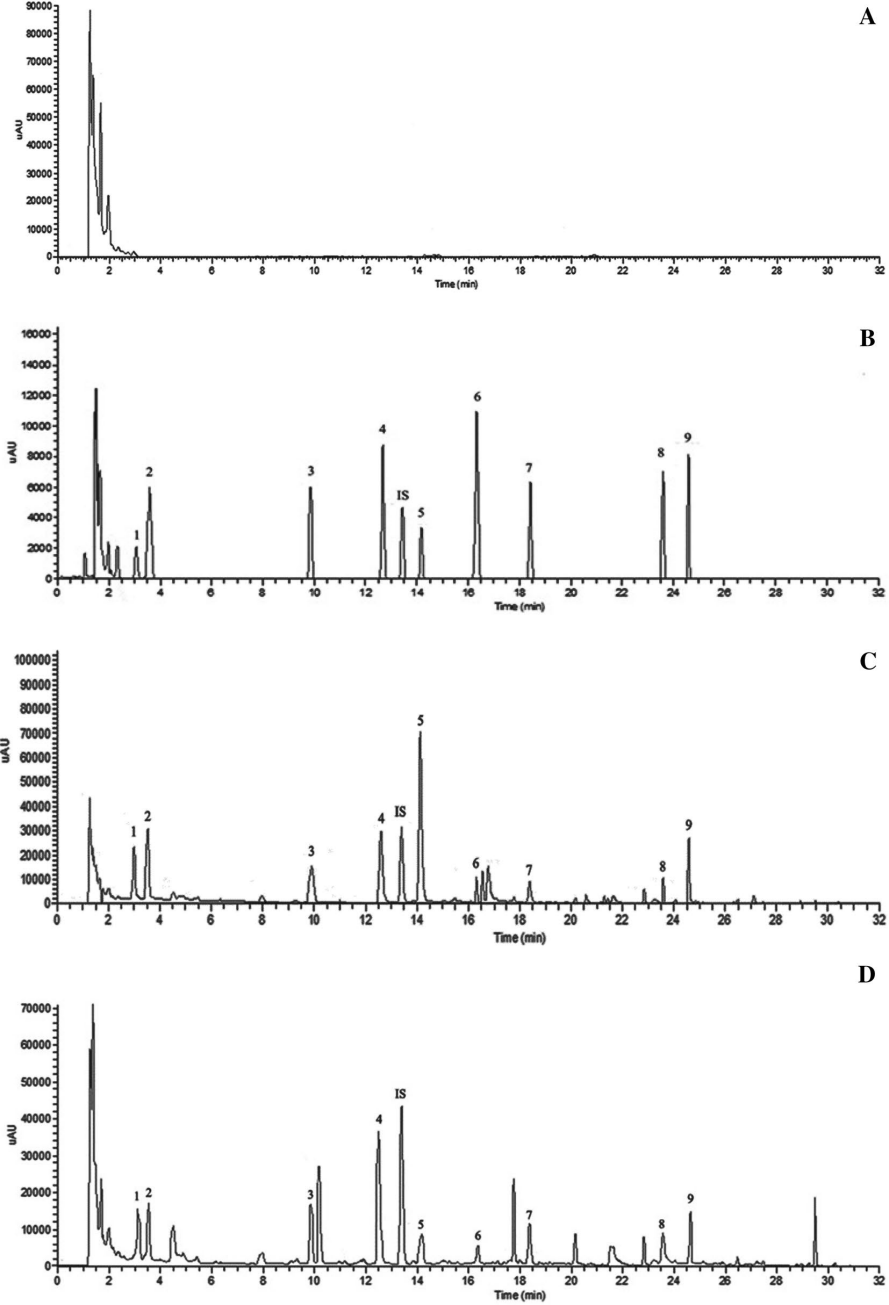
UHPLC chromatograms of rats serum samples: **A** blank serum; **B** blank serum spiked with the nine analytes and IS; **C** serum sample of N-RMC at 1 h; **D** serum sample of BHS-RMC at 1 h; Standards; Standards: (1) 5-HMF; (2) Gallic acid; (3) Oxypaeoniflora; (4) Paeoniflorin; (5) 3, 8-Dihydroxy-2-methylchromone; (6) Benzoic acid; (7) Methyl paraben; (8) Paeonol; (9) Quercetin; (IS) 3-Hydroxy-4-methoxyacetophenone

As shown in Fig. 3, the nine components and IS was not detected in blank serum and could be well separated and detected in reference compounds spiked serum and serum after oral administration of the RMC extract. The calibration curves of each target component displayed linearity over a range of concentration. The analytes had good linearity with correlation coefficients (R^2^ ≥ 0.9907). The results indicated that the method was suitable for quantitative analysis of nine components and accorded with the pharmacokinetic requirements. (Additional file 1: Table S2). The RSD values of precision test ranged from 0.76% to 9.97% for intraday assays and from 0.23% to 8.90% for interday assays. The accuracy ranged from 91.61% to 101.94% for all the QC levels of nine components (Additional file 1: Table S3). The results of stability were in the range of 93.64–101.99% for short-term stability; 91.12–100.24% for freeze–thaw stability and 91.05–100.23% for long-term stability (Additional file 1: Table S4). The extraction recovery was between 64.12% and 79.15% and matrix effect values ranged from 95.95% to 101.82% for all QC levels of nine components (Additional file 1: Table S5). The results indicated that the precision, accuracy, stability and extraction recovery and matrix effect of the analytical method was acceptable in rat serum.

### The TXB2 and 6-Keto-PGF1 in normal and model rats

**Fig. 4.**
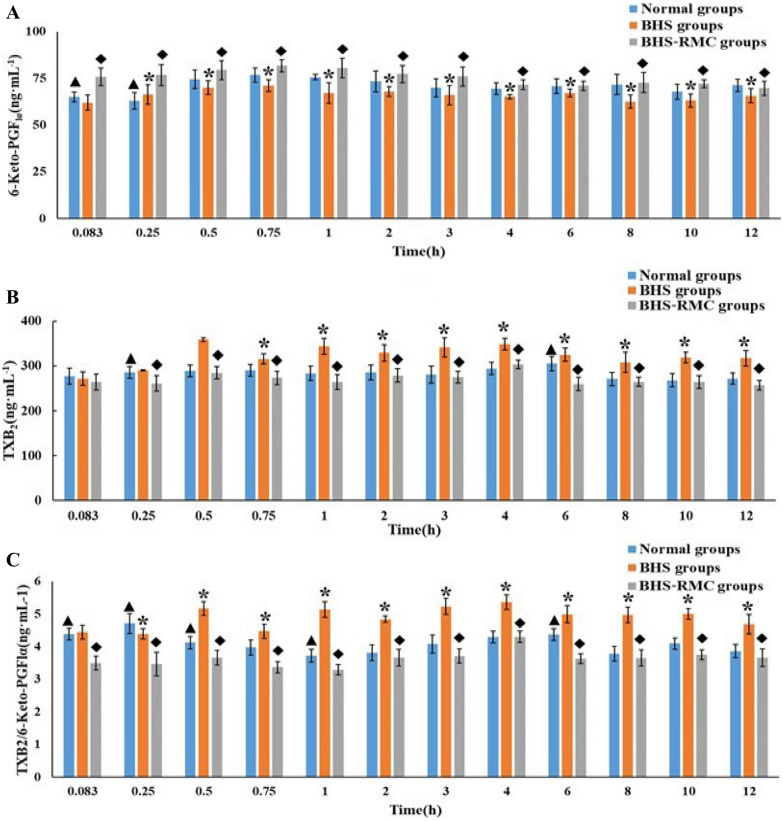
6-Keto-PGFlα (**A**), The TXB2 (**B**) and TXB2/6-Keto-PGFlα (**C**) value of rats in each group at the same time, “*” means there is a significant difference between the BHS model group and the Normal group (*p* < 0.05); “◆” means the BHS group is significantly different from the BHS-RMC group (*p* < 0.05); “▲” means BHS-RMC and Normal Significant difference between groups (*p* < 0.05)

The concentrations of TXB2, 6-Keto-PGFlα and TXB2/6-Keto-PGFlα of the serum in each group rats at the same time point are shown in Fig. 4. The results demonstrated that the 6-Keto-PGFlα value was lower in the BHS model rats comparing to the normal rats after 0.5 h in Fig. 4A, while the TXB2 and TXB2/6-Keto-PGF1 value was higher in the BHS model rats than normal rats in Fig. 4B, C, which implied the BSH model was successful. After oral administration with the RMC extract, it showed a higher level of 6-Keto-PGF1 and a lower level of TXB2 and TXB2/6-Keto-PGF1 in BSH-RMC group as compared with BSH group, and infinitely approaching to the normal group, which confirmed that RMC had a good therapeutic effect on BHS rat.

### Pharmacokinetic study

**Fig. 5:**
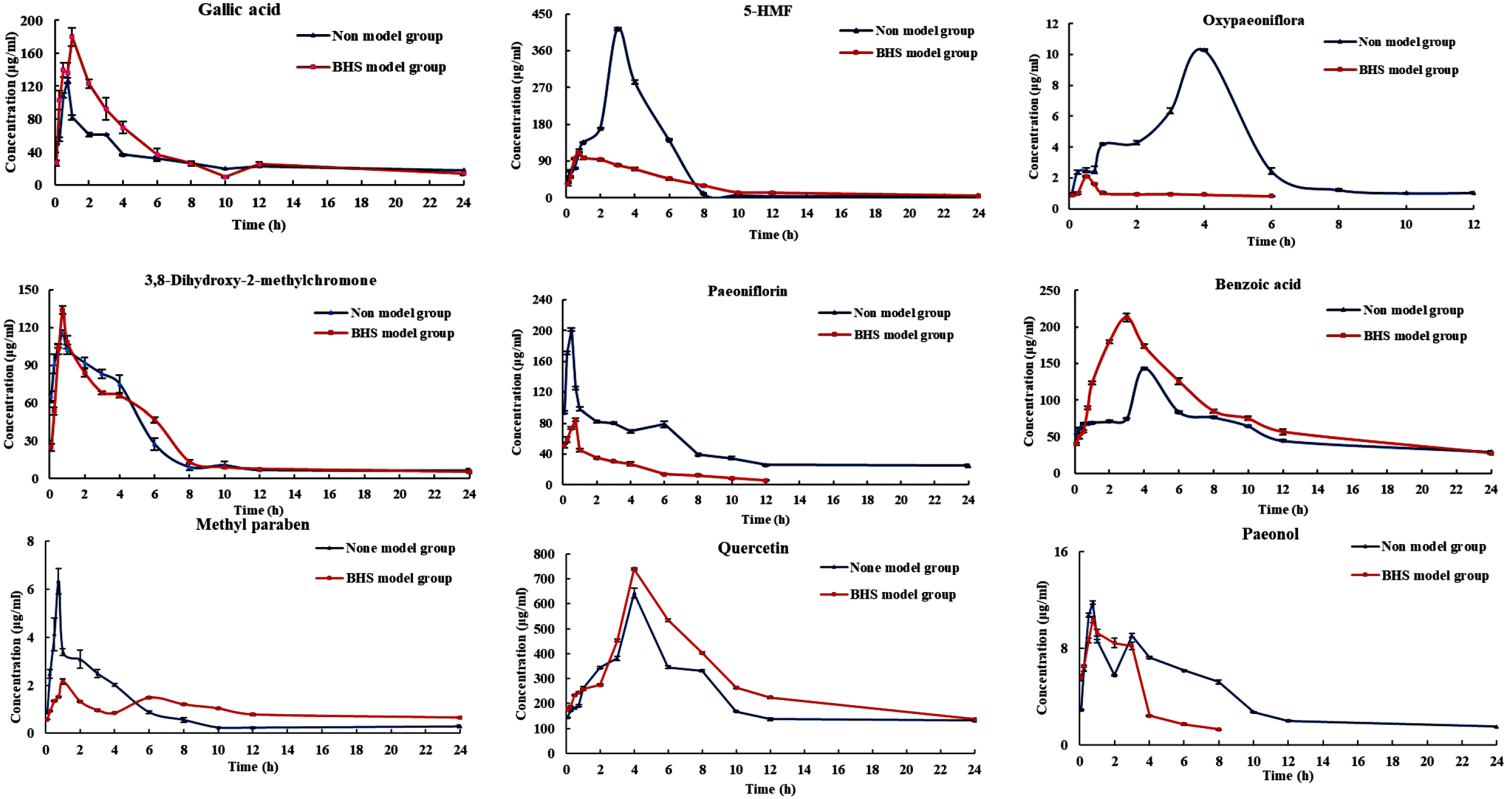
The mean plasma concentration time profiles of Gallic acid, 5-HMF, Oxypaeoniflorin, 3, 8-Dihydroxy-2-methylchromone, Paeoniflorin, Benzoic acid, Methylparaben, Quercetin and Paeonol after oral administration of RMC Extract (n = 6, mean ± SD)

The proposed method was successfully applied to the pharmacokinetic study in BHS groups and normal groups rats after oral administration with the RMC extract. The pharmacokinetic parameters were calculated according to the non-compartmental model. The concentration–time profile of the nine components is illustrated in Fig. 5 and the main pharmacokinetic parameters and significant differences among each group were presented in Table 1. After comparing the pharmacokinetic data of the BHS groups and normal groups rats, it could be concluded that there were significant differences between the pharmacokinetic parameters, such as AUC, Cmax, CL/F (*p* < 0.05).

**Table 1:** The pharmacokinetic parameters of nine components of RMC extracts between N-RMC and BHS-RMC group (n = 6)

Analyte	Group	Pharmacokinetics parameters					
		T_1/2_ (h)	T_max_ (h)	C_max_ (μg/mL)	AUC (μg/mL*h)	MRT (h)	CL/F (mL/min/kg)
Gallic acid	N-RMC	10.76 ± 0.87^◆^	0.75 ± 0.00^◆^	126.96 ± 2.90^◆^	1018.73 ± 26.63	17.30 ± 0.80^◆^	58.93 ± 1.58
	BHS-RMC	6.33 ± 0.45↓	1.00 ± 0.00↑	180.13 ± 11.06↑	1067.98 ± 72.88↑	10.02 ± 0.96↓	56.40 ± 3.79↓
5-HMF	N-RMC	3.20 ± 0.03^◆◆^	3.00 ± 0.00^◆◆^	413.25 ± 2.01^◆◆^	1563.50 ± 4.63^◆◆^	4.55 ± 0.03^◆^	38.38 ± 0.11^◆◆^
	BHS-RMC	7.33 ± 2.10↑	0.75 ± 0.00↓	111.37 ± 7.25↓	740.76 ± 20.26↓	7.47 ± 0.48↑	81.05 ± 2.25↑
Oxypaeoniflorin	N-RMC	3.08 ± 0.98^◆**◆**^	4.00 ± 0.00^◆◆^	10.23 ± 0.11^◆◆^	45.72 ± 1.4^◆^	5.53 ± 0.50^◆◆^	1313.57 ± 40.19^◆^
	BHS-RMC	15.13 ± 2.25↑	0.50 ± 0.00↓	2.09 ± 0.04↓	24.16 ± 3.09↓	21.59 ± 3.42↑	2519.95 ± 343.95↑
3,8-Dihydroxy-2-methylchromone	N-RMC	4.73 ± 0.29^◆◆^	0.75 ± 0.00	115.71 ± 2.12^◆^	660.32 ± 29.92^◆^	6.90 ± 0.68^◆◆^	91.01 ± 4.00^◆◆◆^
	BHS-RMC	19.01 ± 1.78↑	0.75 ± 0.00	134.00 ± 3.26↑	756.93 ± 8.72↑	14.06 ± 1.07↑	79.28 ± 0.92↓
Paeoniflorin	N-RMC	9.75 ± 0.47^◆◆^	0.50 ± 0.00^◆^	201.57 ± 1.86^◆◆^	1449.69 ± 62.31^◆◆^	15.53 ± 0.94^◆◆^	41.45 ± 1.78^◆◆^
	BHS-RMC	3.60 ± 0.31↓	0.75 ± 0.00↑	84.23 ± 1.99↓	298.12 ± 7.60↓	5.06 ± 0.22↓	201.37 ± 5.16↑
Benzoic acid	N-RMC	11.11 ± 1.88	4.00 ± 0.00^◆^	143.65 ± 4.48^◆^	1877.15 ± 216.74^◆^	16.96 ± 2.76^◆^	32.30 ± 3.70^◆^
	BHS-RMC	10.04 ± 0.67↓	3.00 ± 0.00↓	212.92 ± 5.26↑	2319.78 ± 71.29↑	13.26 ± 0.79↓	25.88 ± 0.80↓
Methylparaben	N-RMC	4.93 ± 0.23^◆◆^	0.75 ± 0.00^◆^	6.35 ± 0.53^◆◆^	22.82 ± 1.34^◆^	7.91 ± 0.56^◆◆^	2636.68 ± 156.55^◆^
	BHS-RMC	17.91 ± 0.84↑	1.00 ± 0.00↑	2.16 ± 0.11↓	39.87 ± 1.34↑	27.07 ± 1.16↑	1506.43 ± 50.68↓
Quercetin	N-RMC	9.97 ± 0.35^◆^	4.00 ± 0.00	643.27 ± 19.81^◆^	7338.63 ± 165.50^◆^	16.64 ± 0.56^◆^	8.18 ± 0.18^◆^
	BHS-RMC	15.49 ± 0.54↑	4.00 ± 0.00	739.851 ± 4.17↑	10,036.28 ± 171.6↑	20.45 ± 0.66↑	5.98 ± 0.10↓
Paeonol	N-RMC	7.84 ± 0.08^◆^	0.75 ± 0.00	11.76 ± 0.13^◆^	106.52 ± 1.23^◆◆^	12.11 ± 0.17^◆◆^	563.34 ± 6.49^◆◆^
	BHS-RMC	4.37 ± 0.15↓	0.75 ± 0.00	10.37 ± 0.23↓	45.54 ± 0.83↓	4.63 ± 0.12↓	12,450.58 ± 8613↑

^◆^indicates that there is a significant difference between the N-RMC and the BHS-RMC (*p* < 0.05), and ^◆◆^indicates that the N-RMC and the BHS-RMC are significantly different (*p* < 0.01), ↑ was represented rising in BHS-RMC comparing N-RMC, ↓ was represented declining

### Network analysis

**Fig. 6:**
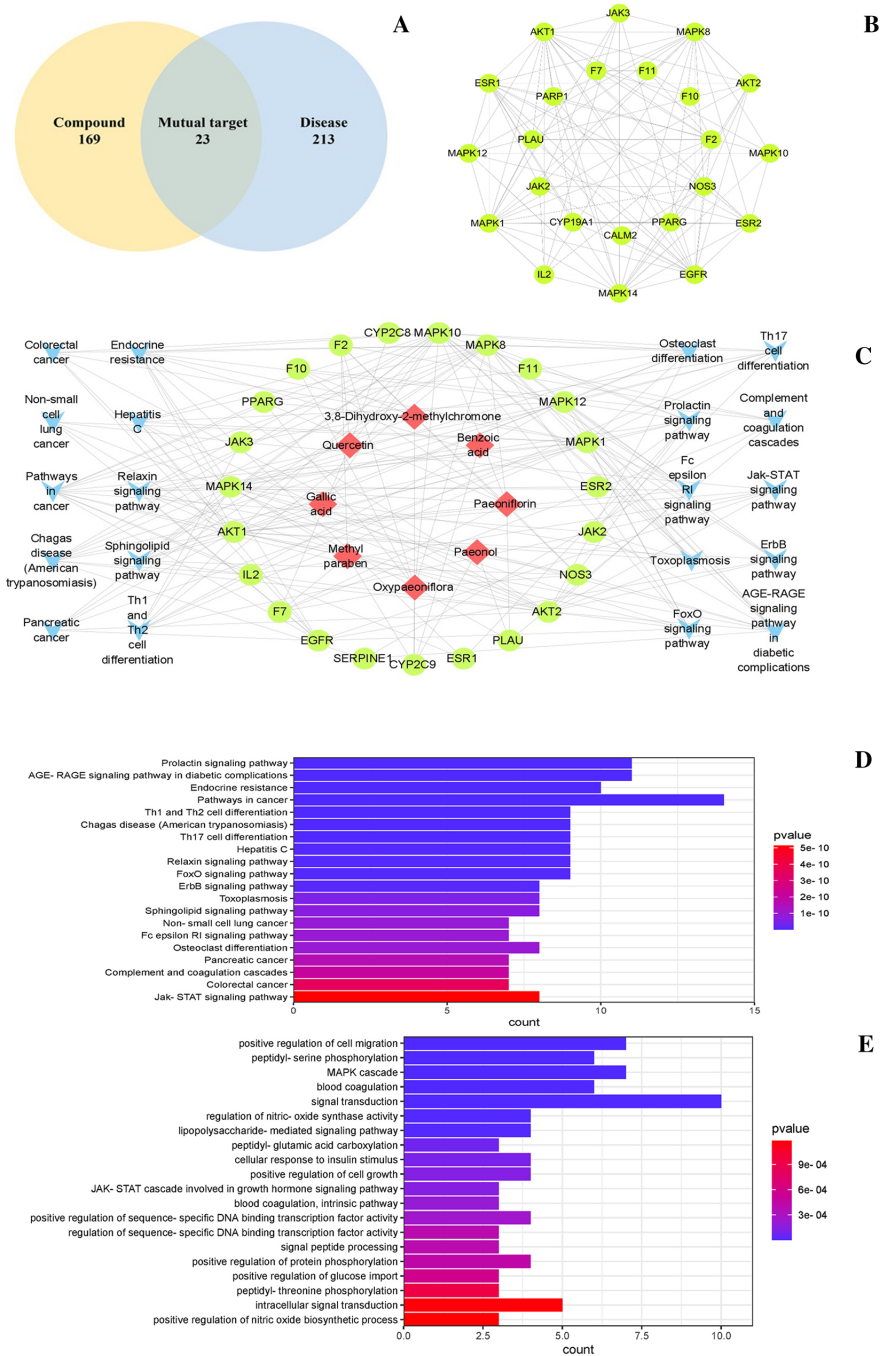
Network analysis of the nine compounds absorbed into the blood in treating BHS syndrome. **A** Targets attribution among the RMC and BHS syndrome. **B** Protein–protein interaction network of hub targets obtained from STRING database and constructed by Cytoscape, target gene indicated by green circle. **C** Top 20 KEGG pathway enrichment entries (*p* < 0.05). **D** Ingredients—target—pathway network. Labels Red diamonds, ingredients; green circles, protein targets; Blue V, Pathway; gray lines, compound–target pathway interactions. **E** Top 20 GO enrichment entries (*p* < 0.05)

As a result, 169 targets of nine absorbed compounds were summarized. Meanwhile, it locked the 213 targets associated with blood-heat and blood stasis syndrome (BHS) (Fig. 6A). Then the overlapping targets were screened from the compound related targets and BHS related targets. Finally, 23 proteins common to compounds and diseases targets were analyzed by PPI (Fig. 6B) (STRING: functional protein association networks) with the score at the high confidence. It is identified as potential targets of RMC for treating BHS Syndrome, which were imported into Cytoscape 3.2.1 for analysis.

A network was constructed to display the compounds–targets interactions (Fig. 6C). As can be seen from the Figure, compounds (paeonol, paeoniflorin, quercetin, oxypaeoniflorin, gallic acid, methyl paraben, benzoic acid and 3, 8-Dihydroxy-2-methylchromone) were shared by 23 proteins that contribute to the regulation of BHS syndrome. They might be the active ingredients in RMC to exert therapeutic effects. Key targets and main components are extracted from the major hub network by defining nodes with degrees higher than the average number of neighbors. Among the 23 proteins, 8 proteins, namely, F2, F10, F7, MAPK14, MAPK10, AKT1, NOS3, PLAU and 7 compounds, such as paeonol, paeoniflorin, quercetin, oxypaeoniflorin, gallic acid, methyl paraben and 3, 8-Dihydroxy-2-methylchromone were found to have the highest degree values, which may be the key treatment targets and active ingredients of RMC for treatment of BHS (Table 2).

**Table 2:** The topological parameters of components and key targets

Name	Degree	Betweenness centrality	Closeness centrality
Oxypaeoniflorin	21	0.56313	0.731707
Paeoniflorin	16	0.260056	0.588235
F7	6	0.110326	0.535714
MAPK14	5	0.093949	0.5
Gallic acid	4	0.072506	0.379747
MAPK10	4	0.059543	0.5
F2	5	0.045091	0.5
NOS3	4	0.030639	0.483871
AKT1	3	0.022278	0.405405
Methyl paraben	4	0.017795	0.4
PLAU	3	0.017271	0.46875
3,8-Dihydroxy-2-methylchromone	5	0.014472	0.38961
F10	3	0.010943	0.46875
PPARG	3	0.010943	0.46875
Quercetin	4	0.009219	0.38961
Paeonol	3	0.005351	0.379747

Based on 23 potential targets, we performed GO and KEGG enrichment analysis. Enrichment of the KEGG pathway could be divided approximately into modules of cancer, anticoagulant and inflammation. (Fig. 6C). In the GO enrichment analysis (Fig. 6E), 23 proteins are mainly involved in the regulation process of transcription factors, which indicated that numerous biologic processes were involved in BHS treatment.

**Fig. 7:**
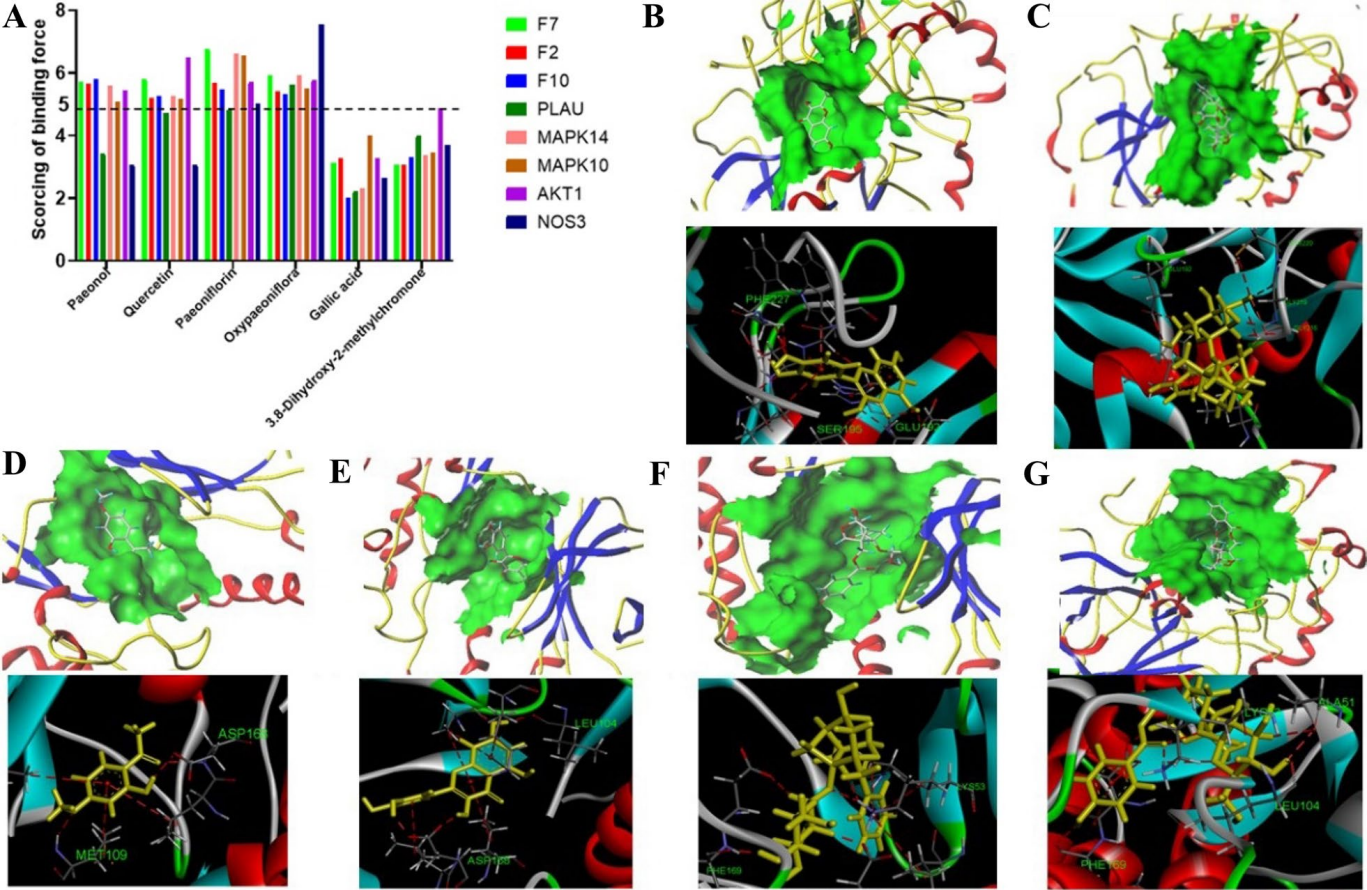
Binding compounds of RMC interacting with target proteins. **A** The scoring of binding force between 6 compounds and 8 proteins. **B**–**G** Binding studies of compounds–targets interactions. Molecules are represented by a yellow stick model, the hydrogen bonds are represented by a red dotted line. quercetin (**B**) paeoniflorin (**C**) within the F2 (3tu7) kinase active site respectively. paeonol (**D**), quercetin (E), Paeoniflorin (**F**), oxypaeoniflora (**G**) within the MAPK14 (ID = 1a9u) kinase active site respectively

Computer-aided interactive molecular docking can be used to predict and analyze the structure–activity relationship between receptor and ligand. The ingredients chosen for molecular docking were based on their high contents analyzed by pharmacological activities screened by network pharmacology. According to the evaluated index of the Surflex-Dock program, the compound has a strong affinity with the target protein when the scoring of binding force is > 5.0. As can be seen from Fig. 7A (molecular docking information results were summarized in the Additional file 1: Table S6), 4 compounds (paeonol, paeoniflorin, quercetin, oxypaeoniflorin) were blinding with 8 hug targets (score > 5), which may be the potential active ingredients of RMC. Among them, F2 and MAPK14 were selected as coagulation and inflammation targets to explore their possible binding interactions. The formation of hydrogen bonds involving the oxygen and the C = O group of the ring, which also makes a contribution to binding. As showed in Fig. 7 (depicted in Additional file 1: Table S7), in the structure of thrombin (F2), quercetin can form a strong conventional hydrogen bond with PHE227, GLU192 and SER195. Additionally, the critical catalytic triads Ser195 were located in the middle of the active site (Fig. 7B). Paeoniflorin has four direct H-bonds to GLY216, GLY219, CYS220 and GLU192 (Fig. 7C). The docked pose of paeonol within MAPK14 showing key hydrogen bond interaction with ASP168 and MET109 (Fig. 7D). Quercetin showing hydrogen bond interaction with ASP168 and LEU104 (Fig. 7E). Compounds showed different hydrogen bonds to the same target, which is due to the different structures of the compounds. Conversely, the binding pattern and alignment of Paeoniflorin and oxypaeoniflorin were found very similar to co-crystal ligand in the binding site because of the similar structure. As can be seen from Fig. 7F, G, Paeoniflorin is bonded with PHE169 and LYS53, whereas the same interaction was also recognized with oxypaeoniflorin.

### Targets validation

**Fig. 8:**
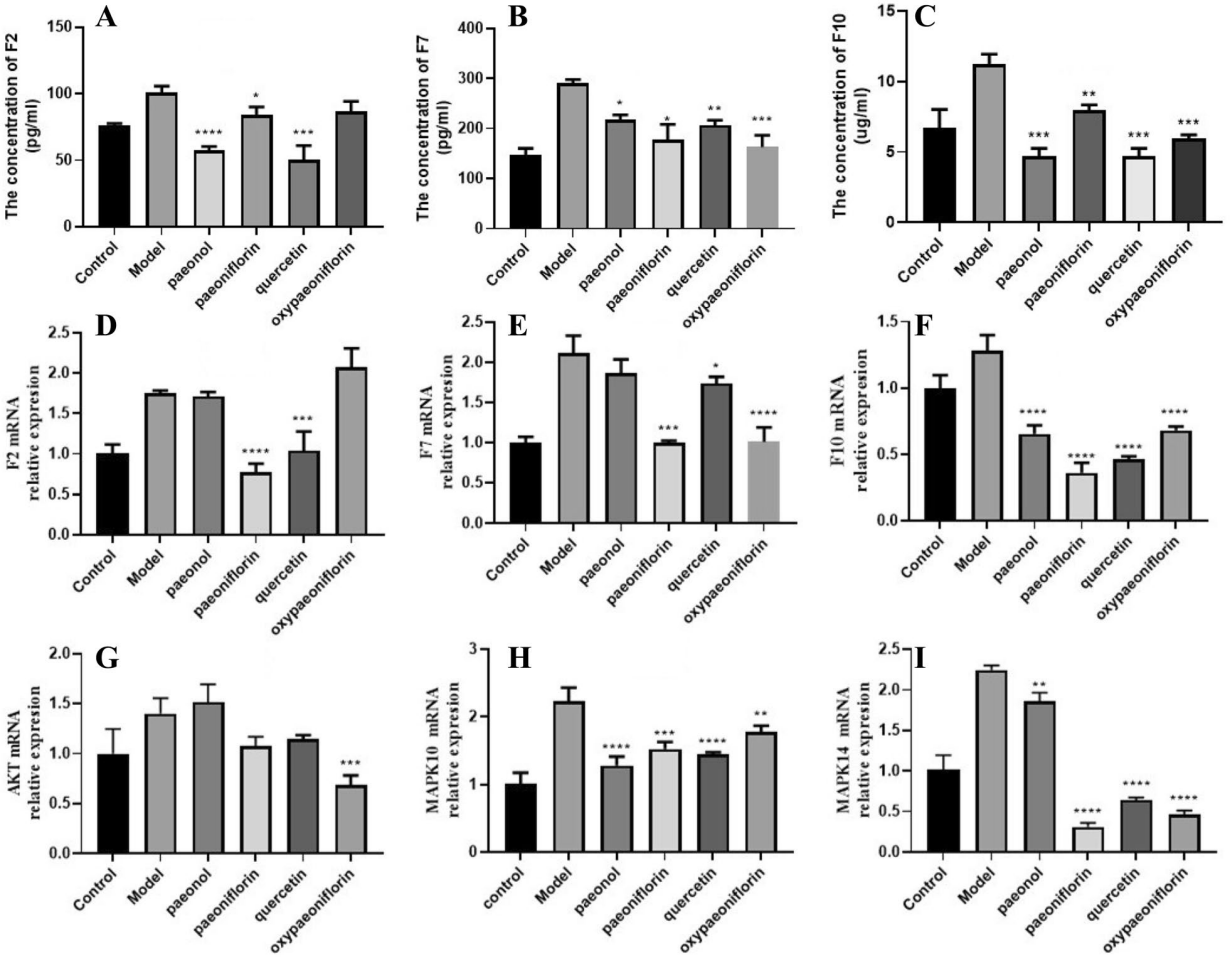
Production of F2 (**A**), F7 (**B**) and F10 (**C**) was determined by ELISA. Protein expression of F2 (**D**), F7 (**E**), F10 (**F**), AKT (**G**), MAPK14 (**H**) and MAPK10 (I) was determined by RT-qPCR. *p < 0.05, ** *p* < 0.01, *** *p* < 0.001, **** *P* < 0.0001 versus Model group

In order to further assess the obtained results in systems pharmacology analysis, we have examined the compound potential activating blood and anti-inflammatory using HUVECs cells induced by TNF-α (25 ng/mL). In particular, we conduct ELISA kit and RT- qPCR analysis for hug targets such as F2, F7 and F10 protein expression to confirm activating blood effects of the 4 compounds (paeonol, paeoniflorin, quercetin and oxypaeoniflorin), as well as RT-qPCR analysis for AKT1, MAPK14 and MAPK10 predicted anti-inflammatory effects of those 4 compounds. As shown in Fig. 8, the levels of F2, F7 and F10 protein in the panel of HUVECs cell lines tested are reported (Fig. 8A–F). We observed that the protein expressions of F2, F7 and F10 protein in HUVECs cells are significantly declined after the treatment of paeonol, quercetin, paeoniflorin and oxypaeoniflorin (excepting oxypaeoniflorin has no effect on F2, paeonol also has no effects on F2 and F7 mRNA expression). Simultaneously, a significant decrease in MAPK14 and MAPK10 mRNA expression was observed after the treatment of those 4 compounds (Fig. 8H–I). Among them, only oxypaeoniflorin could decrease the expression of AKT (Fig. 8G). These data support the in vitro study that the docking prediction is in line with the validated detection by ELISA and RT-PCR, indicating high reliability of our docking.

### Coagulation test

According to the analysis results as mentioned above, these four components (paeoniflorin, oxypaeoniflorin, quercetin and paeonol) were considered to hold a strong activity in their binding to hemostasis-related targets. Therefore, coagulation test was carried out, with APTT, PT and TT used as the analytical indices, as shown in Table 3. The results revealed a significant effect of these four components on prolonging APTT, and has a tendency of prolonging TT, while had no significant difference on PT when comparing with blank. APTT primarily reflects the activity and level of the endogenous coagulation factors [52], which speculated that paeoniflorin, oxypaeoniflorin, quercetin and paeonol could activate blood by inhibiting the endogenous coagulation pathways. These results were also consistent with the findings from HUVECs experiment. But admittedly, the inference on the biological activities of these compounds still needs to be further verified in more in vivo studies.

**Table 3:** The results of coagulation tests of four potential active compounds (n = 3)

No	C (μg/mL)	APTT(s)	PT(s)	TT(s)
Blank	–	13.63 ± 0.49	8.07 ± 0.42	30.00 ± 1.55
Paeoniflorin	250	15.07 ± 0.06^**^	8.37 ± 0.42	31.20 ± 0.92
Oxypaeoniflorin	500	15.63 ± 0.45^****^	7.87 ± 0.31	30.90 ± 1.14
Quercetin	1	15.2 ± 0.387^***^	7.97 ± 0.15	31.03 ± 0.68
Paeonol	50	17.23 ± 0.35^****^	8.47 ± 0.40	30.93 ± 0.31

^*^indicates that there is a significant difference comparing with Blank (*p* < 0.05), ^**^ (*p* < 0.01), ^***^(*p* < 0.001), ^*****^(*p* < 0.0001)

## Discussion

TXB2 and 6-Keto- PGFlα are commonly used as vivo indexes in studying the drug hemostasis mechanism. The values of TXB2, 6-Keto-PGFlα and TXB2/6-Keto-PGFlα between BHS and Normal group, BHS-RMC and BHS model group are significantly different (*p* < 0.05). The TXB2 and TXB2/6-Keto-PGFlα value of the BHS model group are higher than normal group, while the 6-Keto-PGFlα value is lower in normal group after 0.5 h, which indicated that the BHS model is successful. However, after administration, BHS-RMC group showed lower level of TXB2 and TXB2/6-Keto–PGFlα and higher level of 6-Keto-PGFlα as compared to BHS model group. Simultaneously, there was no significant difference between the normal group and BHS-RMC group (Fig. 4). The results showed that RMC has a better therapeutic effect on BHS syndrome and can correct the pathological state to be close to the normal state.

The nine components were measurable in plasma after oral administration RMC extract. The nine compounds pharmacokinetics parameters varied in normal and BHS rats considerably (Table 1), which revealed that the pathological state could influence the pharmacokinetic characteristics of compounds. The AUC and C_max_ of 3, 8-dihydroxy-2-methylchromone, benzoic acid, quercetin and gallic acid in BHS-RMC were greater than N-RMC (AUC increased by 15%, 23%, 36%, 5% and 74%; C_max_ increased by 16%, 49%, 15% and 42% respectively), while their CL/F value is significantly lower, which indicated that these four components had the higher exposure and bioavailability as well as slower elimination rate in BHS rats than normal rats. In contrast, 5-HMF, oxypaeoniflorin, paeoniflorin, and paeonol showed smaller AUC0-t and Cmax in BHS-RMC group, while CL/F value increased. Significantly, it indicated that these four components showed lower exposure and bioavailability as well as fast metabolism in BHS-RMC as compared to those in N-RMC, which revealed that the pathological state could influence the pharmacokinetic characteristics of compounds.

In addition, it was noticeable that oxypaeoniflorin was not detected in BHS-RMC rats after 6 h. (Fig. 5). Similarly, paeonol and paeoniflorin are not detected after 8 h and 12 h, respectively, which indicated that pathological state accelerated the metabolic process of the drug, resulting changes in the body's absorption, distribution, metabolism and excretion of these three components. It was also found that the plasma profile of paeonol presented bimodal (double peak) phenomenon after oral administration of RMC extract in both normal and model rats, which are consistent with those reported in the literature [53, 54]. According to reports, paeonol had a strong first-pass effect, metabolized and excreted quickly in the body, which resulted in low bioavailability. Furthermore, the bioactivity of paeonol metabolites that had been confirmed has pharmacological activity, which suggested that the absorption and paeonol were accelerated under pathological conditions, and its metabolites were quickly absorbed into the blood to play a therapeutic role [54, 55]. As for paeoniflorin and oxypaeoniflorin, studies had shown that they were generally enzymatically hydrolyzed into aglycones by intestinal flora to exert their therapeutic effects, which suggested that the intestinal flora had changed under the pathological state, thereby accelerating the metabolism of paeoniflorin and oxypaeoniflorin into aglycones to quickly exert curative effects [56–59], which resulted the low bioavailability and high CL/F value under pathological conditions.

However, Cortex Moutan Carbon, the Processed product of Moutan Cortex (PMC), had the opposite effect of cooling blood and hemostatic. Our last article research found that PMC may promote the absorption of 5-HMF and inhibit oxypaeoniflorin, quercetin and 3, 8-Dihydroxy-2- methylchromone absorption in Blood-Heat and Hemorrhage Syndrome Model rat (BHH) as compared with the normal rats [37]. The absorption of three components (3, 8-dihydroxy-2-methylchromone, benzoic acid, quercetin) was promoted and 5-HMF was inhibited in BHS-RMC. These revealed the opposite effective and processing mechanism of RMC.

Network analysis and molecular docking showed that the 4 compounds absorbed into the blood were capable of binding with F2, F10, F7, AKT1, MAPK14, MAPK10, PLAU and NOS3. According to the literature, F2, F7, F10 involved the coagulation system, which were directly involved in the mechanism of blood coagulation [42]. Tumor necrosis factor (TNF-α) could attack endothelial cells, which damaged vascular endothelial cells and caused abnormal function of coagulation factors [60]. HUVECs verification experiment had found that 4 compounds (paeonol, quercetin, paeoniflorin and oxypaeoniflorin) could significantly decline the content of F2, F7 or F10 protein in TNF-α induced HUVECs. Coagulation test revealed that four components could activate blood circulation by prolonging APTT. It has been previously reported that the coagulation system and the inflammatory system interact with each other. Not only does inflammation led to the activation of coagulation, but coagulation in turn affects inflammation [61]. MAPK10 and MAPK14 are members of the MAPK family that is widely involved in the inflammatory response and platelet activation. It plays a key role in the inflammatory response and thrombosis [62, 63]. Akt could be activated by a variety of platelet proteins, thus played an important role in platelet aggregation and hemostasis [64]. Those 4 compounds, paeonol, quercetin, paeoniflorin and oxypaeoniflorin may inhibit the expression of MAPK14 and MAPK10 to exert anti-inflammatory effect. In addition, oxypaeoniflorin could reduce the expression of AKT. According to the literatures, Paeoniflorin could inhibit the expression and secretion of inflammation-related proteins and chemokines by regulating p-MAPK. In addition, it could reduce platelet aggregation rate [65]. Paeonol and its metabolites had anti-inflammatory and antioxidant effects by blocking the MAPK/ERK/p38 signaling pathway [66]. Quercetin inhibited TNF-α induced MAPK pathway activation, also had anti-angiogenic effects, which inhibited expression of proinflammatory factors and endothelin1 [67–69]. These datas suggested that paeonol, quercetin, paeoniflorin and oxypaeoniflorin may be the active components of RMC for cooling and promoting blood circulation. And the mechanism of RMC against BHS syndrome may be acts on MAPK14 and MAPK10, which inhibited inflammatory pathways to regulate the expression of inflammatory, as well as interacted with F2, F10 and F7 regulated coagulation cascades pathway, which improve blood circulation, exerting anticoagulation and antiplatelet aggregation effects.

## Conclusion

A network analysis integrated pharmacokinetics strategy was used to study the mechanism of action of nine components absorbed into blood after oral administration of RMC extracts in BHS and normal rats. The experimental results indicated that the mechanism of RMC for promoting blood circulation and cooling blood may be the regulation of the target genes, namely, F2, F10, F7, MAPK14 and MAPK10 through coagulation and inflammation pathways. It improves microcirculation, anti-inflammatory and inhibit platelet aggregation rate. The proposed pharmacokinetics integrated network analysis strategy in this experiment provides a combined method to systematically explore the absorption mechanism of multi-component drugs in the blood, clarifying the role of the nine components of RMC in the body. It lays the foundation for revealing the mechanism of RMC treatment of BHS.

## Supplementary Information

**Additional file 1: Table S1**. The primers used in RT-qPCR . **Table S2.** Calibration curves, linear range of nine compounds. **Table S3.** Intra-day and inter-day accuracy and precision of nine compounds in rat serum. **Table S4.** Stability of nine compounds in rat serum (mean ± SD，n=5). **Table S5.** Extraction recovery and Matrix effect of nine compounds in rat serum (mean ± SD，n=5). **Table S6**. The molecular docking information results. **Table S7. **Binding sites of the compounds and the interaction. **Figure S1.** Inhibitory effect of 4 compounds on HUVEC(MTT).

## Data Availability

The datasets used and/or analyzed during the current study are available from the corresponding author upon reasonable request.

## Abbreviations

RMC: Raw Moutan Cortex
BHS: Blood-heat and blood stasis syndrome
UHPLC-DAD: Ultra-High performance Liquid Chromatography coupled with Diode Array Detector
HUVECs: Human umbilical vein endothelial cells
TCMSP: Traditional Chinese Medicine Systems Pharmacology Database and Analysis Platform
TTD: Therapeutic target database
PDB: Protein data bank
PPI: Protein–Protein Interactions
KEGG: Kyoto encyclopedia of genes and genomes
GO: Genome ontology
TT: Thrombin time
PT: Prothrombin time
APTT: Activated partial thromboplastin time
