# Supplementary material for: Integrating pharmacokinetics and network analysis to investigate the mechanism of Moutan Cortex in blood-heat and blood stasis syndrome
Source: Chin Med. 2022 Sep 14;17:107. doi: 10.1186/s13020-022-00657-w (PMC9476706; doi:10.1186/s13020-022-00657-w)

**Table S1**. The primers used in RT-qPCR were shown in Table1.

| **Primer** | **sequence (5' to 3')** |
| --- | --- |
| GAPDH | F : ACGGATTTGGTCGTATTGG |
|  | R : TCCCGTTCTCAGCCTTG |
| MAPK14 | F : TTTGCTGGCTCTTGGAAC |
|  | R : CGATCTCCCTGCACCTT |
| MAPK10 | F : AGCAATAATCAGGCTTCCC |
|  | R : TCATCAACCATCCACTTCC |
| AKT1 | F : TGTGAAGGAGGGTTGGCT |
|  | R : GCGCCACAGAGAAGTTGTT |
| F10 | F : CGGCTACGACACCAAGC |
|  | R : ACTTGAGGAAGGCGGTGA |
| F2 | F : GCAAGCACCAGGACTTCA |
|  | R : GAGGTCGCAGTACCCAAAG |
| F7 | F： CTGAAGGCGGTTGTTTAGC |
|  | R： GGAAGCAGGTGGGGAATA |

**Table S2. Calibration curves, linear range of nine compounds.**

| **Compounds** | **Calibration Curves** | **R^2^** | **Linear range**  **(μg/ml)** | **LLOQ**  **(μg/ml)** |
| --- | --- | --- | --- | --- |
| 5-HMF | Y=0.0186x + 0.3545 | 0.9977 | 3.42~489.21 | 3.42 |
| Gallic acid | Y=0.0225x + 0.1842 | 0.9947 | 5.41~217.68 | 5.41 |
| Oxypaeoniflorin | Y=0.0181x + 0.2396 | 0.9939 | 0.58~11.89 | 0.58 |
| Paeoniflorin | Y=0.002x + 0.0016 | 0.9968 | 3.78~252.73 | 3.78 |
| 3, 8-Dihydroxy-2-methylchromone | Y=0.0228x + 0.0314 | 0.9980 | 3.39~164.31 | 3.39 |
| Benzoic acid | Y = 0.0063x - 0.0248 | 0.9925 | 24.78~269.98 | 24.78 |
| Methyl paraben | Y=0.0858x + 0.0162 | 0.9915 | 0.15~8.04 | 0.15 |
| Paeonol | Y=0.0211x - 0.0143 | 0.9977 | 1.69~14.52 | 1.69 |
| Quercetin | Y=0.0502x - 1.6608 | 0.9907 | 107.66~895.54 | 107.66 |

**Table S3 Intra-day and inter-day accuracy and precision of nine compounds in rat serum**

| **Compounds** | **Spiked**  **concentration(μg/ml)** | **Intraday** | | | **Interday** | | |
| --- | --- | --- | --- | --- | --- | --- | --- |
|  |  | **Measured**  **concentration (μg/ml, mean ± SD)** | **Accuracy (%)** | **Precision RSD (%)** | **Measured**  **concentration (μg/ml, mean ± SD)** | **Accuracy (%)** | **Precision RSD (%)** |
| 5-HMF | 405.90 | 397.29±1.45 | 97.88 | 1.23 | 398.09±9.56 | 98.08 | 6.10 |
|  | 110.70 | 102.22±1.87 | 92.34 | 2.76 | 103.93±2.20 | 93.88 | 5.42 |
|  | 18.45 | 17.22±1.36 | 93.33 | 3.45 | 17.25±1.14 | 93.50 | 6.23 |
| Gallic acid | 186.75 | 181.97±5.12 | 97.44 | 2.12 | 182.45±4.20 | 97.70 | 1.23 |
|  | 93.38 | 89.04±3.45 | 95.35 | 2.56 | 90.96±4.21 | 97.41 | 2.23 |
|  | 20.75 | 19.01±0.56 | 91.61 | 1.75 | 20.06±0.52 | 96.67 | 1.23 |
| Oxypaeoniflorin | 6.40 | 5.93±0.39 | 92.66 | 3.23 | 6.02±0.24 | 94.06 | 1.89 |
|  | 3.20 | 2.95±0.18 | 92.19 | 1.85 | 2.98±0.12 | 93.13 | 2.21 |
|  | 0.96 | 0.89±0.02 | 92.71 | 0.76 | 0.91±0.36 | 94.79 | 3.89 |
| Paeoniflorin | 145.28 | 138.74±3.85 | 95.50 | 5.45 | 139.29±6.42 | 95.88 | 3.25 |
|  | 67.05 | 65.78±1.59 | 98.11 | 4.52 | 66.68±2.23 | 99.45 | 3.45 |
|  | 16.76 | 16.00±0.81 | 89.50 | 4.05 | 15.76±0.87 | 94.03 | 6.21 |
| 3,8-Dihydroxy-2  -methylchromone | 129.38 | 123.71±1.08 | 95.62 | 1.92 | 125.30±1.63 | 96.85 | 2.23 |
|  | 73.13 | 67.71±0.24 | 92.59 | 0.63 | 69.49±0.36 | 95.02 | 0.23 |
|  | 16.88 | 15.04±0.15 | 95.02 | 2.12 | 15.12±2.18 | 89.57 | 6.71 |
| Benzoic acid | 243.75 | 234.56±2.25 | 96.23 | 1.12 | 136.39±3.67 | 100.95 | 3.64 |
|  | 97.50 | 93.56±2.43 | 95.96 | 3.30 | 96.66±2.73 | 99.14 | 3.12 |
|  | 40.63 | 38.04±0.25 | 93.63 | 4.93 | 39.01±0.62 | 96.01 | 5.12 |
| Methyl paraben | 6.50 | 6.29±0.19 | 96.77 | 3.29 | 6.23±0.18 | 95.85 | 1.01 |
|  | 3.90 | 3.60±0.05 | 92.31 | 1.55 | 3.71±0.13 | 95.13 | 3.12 |
|  | 1.30 | 1.21±0.02 | 93.08 | 8.82 | 1.21±0.02 | 93.08 | 1.23 |
| Paeonol | 12.05 | 11.48±1.37 | 95.27 | 4.48 | 11.37±0.23 | 94.36 | 1.02 |
|  | 7.23 | 6.88±0.33 | 95.16 | 2.04 | 6.63±2.23 | 91.70 | 1.09 |
|  | 2.41 | 2.25±0.17 | 93.36 | 7.95 | 2.36±0.18 | 97.93 | 2.90 |
| Quercetin | 715.50 | 698.61±15.88 | 97.64 | 3.80 | 693.58±11.45 | 101.94 | 8.90 |
|  | 357.75 | 347.97±2.45 | 97.27 | 1.21 | 349.95±8.23 | 97.82 | 3.09 |
|  | 107.33 | 101.28±1.89 | 94.36 | 9.97 | 102.39±1.64 | 95.40 | 3.32 |

**Table S4. Stability of nine compounds in rat serum (mean ± SD，n=5).**

| **Compounds** | **Spiked**  **Concentration (μg/ml)** | **Short-term stability (%)** | **Freeze-thaw stability (%)** | **Long-term stability (%)** |
| --- | --- | --- | --- | --- |
| 5-HMF | 405.90 | 98.49±2.33 | 92.50±1.32 | 91.05±1.21 |
|  | 110.70 | 96.62±2.12 | 95.44±2.21 | 95.2 9±4.89 |
|  | 18.45 | 97.06±3.20 | 96.33±2.31 | 95.24±2.01 |
| Gallic acid | 186.75 | 94.25±1.41 | 93.00±1.25 | 93.26±1.52 |
|  | 93.38 | 97.26±1.56 | 96.72±1.63 | 95.73±2.42 |
|  | 20.75 | 98.70±2.56 | 98.02±3.02 | 100.23±2.30 |
| Oxypaeoniflorin | 6.40 | 97.81±1.33 | 95.51±2.14 | 93.42±2.41 |
|  | 3.20 | 97.86±2.02 | 96.22±2.31 | 96.23±4.30 |
|  | 0.96 | 98.45±1.07 | 98.54±3.32 | 97.41±2.75 |
| Paeoniflorin | 145.28 | 93.88±3.16 | 91.12±3.88 | 58.83±1.56 |
|  | 67.05 | 95.55±2.89 | 94.39±1.02 | 95.19±2.40 |
|  | 16.76 | 97.66±3.56 | 95.27±0.75 | 96.05±3.15 |
| 3,8-Dihydroxy-2-methylchromone | 129.38 | 101.99±0.28 | 100.24±2.23 | 100.14±2.04 |
|  | 73.13 | 94.32±2.63 | 95.21±1.86 | 97.52±1.56 |
|  | 16.88 | 98.79±2.30 | 96.15±1.38 | 93.29±3.22 |
| Benzoic acid | 243.75 | 95.72±3.71 | 93.60±0.86 | 91.69±1.02 |
|  | 97.50 | 96.05±4.02 | 92.39±2.01 | 91.24±4.23 |
|  | 40.63 | 96.83±5.63 | 92.18±2.62 | 88.94±1.21 |
| Methyl paraben | 6.50 | 93.64±1.93 | 90.19±2.20 | 99.94±1.26 |
|  | 3.90 | 99.13±1.23 | 99.11±1.28 | 98.14±1.23 |
|  | 1.30 | 99.09±7.25 | 98.91±4.19 | 94.26±2.36 |
| Paeonol | 12.05 | 100.63±2.38 | 100.02±2.16 | 98.12±4.12 |
|  | 7.23 | 96.10±3.02 | 95.16±2.07 | 94.40±2.09 |
|  | 2.41 | 98.96±2.00 | 97.06±2.17 | 92.82±5.85 |
| Quercetin | 715.50 | 96.69±1.54 | 95.36±1.22 | 94.59±2.51 |
|  | 357.75 | 97.53±5.23 | 96.40±1.24 | 95.53±0986 |
|  | 107.33 | 99.63±3.20 | 98.04±3.55 | 98.65±2.20 |

**Table S5. Extraction recovery and Matrix effect of nine compounds in rat serum (mean ± SD，n=5)**

| Compounds | Spiked  Concentration (μg/ml) | Extraction recovery（%） | RSD（%） | Matrix effect（%） | RSD（%） |
| --- | --- | --- | --- | --- | --- |
| 5-HMF | 405.90 | 79.15±1.15 | 10.81 | 99.65 | 6.27 |
|  | 110.70 | 76.29±1.53 | 6.92 | 99.26 | 9.15 |
|  | 18.45 | 75.22±1.22 | 7.55 | 98.00 | 5.76 |
| Gallic acid | 186.75 | 74.82±1.00 | 6.74 | 97.18 | 5.76 |
|  | 93.38 | 72.41±0.55 | 2.46 | 99.91 | 5.82 |
|  | 20.75 | 68.64±1.51 | 2.25 | 100.01 | 2.91 |
| Oxypaeoniflorin | 6.40 | 72.37±2.13 | 12.38 | 97.62 | 9.27 |
|  | 3.20 | 72.93±0.82 | 6.15 | 101.21 | 5.75 |
|  | 0.96 | 76.42±1.09 | 3.45 | 98.56 | 5.65 |
| Paeoniflorin | 145.28 | 66.76±1.55 | 3.65 | 99.55 | 9.56 |
|  | 67.05 | 64.20±1.22 | 11.12 | 99.17 | 8.60 |
|  | 16.76 | 65.89±2.56 | 12.55 | 99.15 | 2.59 |
| 3,8-Dihydroxy-2-methylchromone | 129.38 | 75.97±1.23 | 3.12 | 98.43 | 3.42 |
|  | 73.13 | 74.48±1.99 | 9.46 | 98.21 | 7.29 |
|  | 16.88 | 72.12±1.22 | 5.76 | 99.94 | 5.28 |
| Benzoic acid | 243.75 | 75.88±1.89 | 10.27 | 97.45 | 5.21 |
|  | 97.50 | 74.56±2.71 | 3.21 | 97.18 | 7.72 |
|  | 40.63 | 69.82±4.91 | 9.11 | 98.92 | 7.65 |
| Methyl paraben | 6.50 | 77.08±1.84 | 11.30 | 97.78 | 9.98 |
|  | 3.90 | 75.26±1.24 | 9.99 | 98.42 | 5.23 |
|  | 1.30 | 78.88±3.47 | 8.13 | 95.95 | 3.50 |
| Paeonol | 12.05 | 68.76±0.91 | 7.92 | 98.97 | 5.20 |
|  | 7.23 | 64.12±2.87 | 3.01 | 98.32 | 6.76 |
|  | 2.41 | 65.87±1.96 | 7.71 | 101.20 | 5.28 |
| Quercetin | 715.50 | 67.62±0.71 | 10.27 | 98.96 | 11.11 |
|  | 357.75 | 66.35±1.71 | 4.95 | 99.80 | 9.04 |
|  | 107.33 | 65.45±1.67 | 7.89 | 98.81 | 2.24 |

**Table S6**. The molecular docking information results

| Compounds | protein | Total_Score | Crash | Polar | Total Interaction Energy (kcal/mol) | Residue |
| --- | --- | --- | --- | --- | --- | --- |
| Paeoniflorin | F7 | 6.76 | -2.22 | 2.02 | -13.23 | H_HIS57, H_SER190, H_LYS192,H_SER195,  H_SER214,H_GLY216,H_CYS22. |
| Oxypaeoniflora |  | 5.92 | -0.65 | 3.96 | -32.11 | H_TYR94, H_GLY97,H_SER190,H_CYS191,H_LYS192,  H_SER195,H_TRP215,H_GLY216,H_GLY219,H_GLY226. |
| Paeonol |  | 5.7 | -1.36 | 1.97 | -14.02 | H_GLY216,H_CYS220,H_ALA221A, H_VAL227. |
| Quercetin |  | 5.81 | -1.37 | 0.45 | -17.24 | H_SER190, H_LYS192, H_VAL213,H_TRP215,H_GLY216,  H_GLN217,H_GLY219,H_CYS220,H_GLY226,H_VAL227. |
| Gallic acid |  | 3.12 | -0.80 | 5.56 | -35.45 | H_HIS57,H_CYS58,H_LYS60A,H_LYS192,H_SER195. |
| 3,8-Dihydroxy-2-methylchromone |  | 3.08 | -1.06 | 0.02 | -0.46 | H_SER195, H_VAL213,H_TRP215,H_GLY216,H_GLN217,  H_CYS220. |
|  |  |  |  |  |  |  |
| Paeoniflorin | F2 | 5.68 | -2.76 | 4.58 | -20.47 | H_HIS57, H_TYR60A, H_TRP60D, H_LEU99, H_ALA190,H_GLU192,H_SER195,H_GLY216. |
| Oxypaeoniflora |  | 5.42 | -2.07 | 2.80 | -50.98 | H_TRP60D, H_LEU99, H_GLU192, H_GLY216, HGLY219,H_CYS220. |
| Paeonol |  | 5.66 | -1.79 | 3.97 | 5.39 | H_ALA190, H_CYS191, H_GLY216, H_CYS220,  H_GLY226. |
| Quercetin |  | 5.21 | -2.00 | 2.56 | 4.18 | H_GLU192, H_SER195,H_VAL213,H_SER214,H_TRP215,  H_GLY216,H_GLY226,H_PHE227. |
| Gallic acid |  | 3.27 | -0.68 | 1.89 | -1.77 | H_VAL213,H_SER214,H_TRP215,H_GLY216,,H_GLY219,  H_CYS220,H_GLY226,H_PHE227,H_TYR228. |
| 3,8-Dihydroxy-2-methylchromone |  | 3.08 | -0.21 | 3.05 | -38.89 | H_TRP60D,H_GLU192,H_SER195,H_GLY216. |
|  |  |  |  |  |  |  |
| Paeoniflorin | F10 | 5.33 | -4.42 | 6.62 | -29.52 | A_HIS57,A_ASP102,A_CYS191,A_GLN192, A_GLY193,A_SER195,A_GLY216,A_GLY218. |
| Oxypaeoniflora |  | 5.46 | -1.63 | 3.83 | -13.67 | A_GLU97,A_THR98,A_TYR99,A_PHE174,A_ILE175,  A_TRP215. |
| Paeonol |  | 5.79 | -0.65 | 0.49 | -8.99 | A_ASP189,A_ALA190,A_GLN192,A_SER195,A_TRP215,  A_CYS220. |
| Quercetin |  | 5.27 | -0.64 | 2.05 | -35.38 | A_GLU97, A_THR98, A_TYR99, A_PHE174,A_TRP215. |
| Gallic acid |  | 2.01 | -0.64 | 2.05 | -6.95 | A_GLU97, A_THR98, A_TYR99, A_PHE174,A_ILE175,  A_TRP215. |
| 3,8-Dihydroxy-2-methylchromone |  | 3.32 | -0.25 | 0.94 | -11.03 | A_THR98, A_PHE174, A_TRP215. |
|  |  |  |  |  |  |  |
| Paeoniflorin | MAPK14 | 6.62 | -2.48 | 2.21 | -5.45 | A_TYR35, A_ALA51, A_LYS53, A_GLU71, A_LEU104,  A_ASP168, A_PHE169. |
| Oxypaeoniflora |  | 5.92 | -3.55 | 2.90 | 3.07 | A_ALA51, A_LYS53, A_ILE84, A_LEU104, A_MET109,  A_LEU167, A_ASP168, A_PHE169. |
| Paeonol |  | 5.60 | -0.39 | 3.52 | -18.84 | A_ALA51, A_MET109, A_LEU167, A_ASP168. |
| Quercetin |  | 5.39 | -1.60 | 3.37 | 1.01 | A_ALA51, A_LYS53, A_ILE84, A_LEU104, A_LEU167,  A_ASP168. |
| Gallic acid |  | 4.57 | 0.52 | 5.78 | -59.58 | A_LYS53, A_HIS107, A_MET109, A_ASP168. |
| 3,8-Dihydroxy-2-methylchromone |  | 3.98 | -1.29 | 1.18 | 0.52 | A_VAL38, A_ALA51, A_LYS53, A_LEU75, A_LEU104. |
|  |  |  |  |  |  |  |
| Paeoniflorin | PLAU | 4.81 | -0.74 | 2.67 | -21.09 | A_CYS191, A_GLN192, A_GLY193, A_SER195, |
| Oxypaeoniflora |  | 5.62 | -1.56 | 3.34 | -14.50 | A_CYS191.A_GLN192,A_GLY193,A_SER195,  A_GLY216,A_GLY219, A_CYS220 |
| Paeonol |  | 3.43 | -3.87 | 2.90 | -36.58 | A_CYS42, A_HIS57,A_LEU97B,A_SER190,A_GLN192,  A_SER214,A_TRP215,A_GLY216,A_GLY226. |
| Quercetin |  | 4.72 | -1.50 | 1.61 | -2.60 | A_VAL41,A_HIS57,A_GLY193. |
| Gallic acid |  | 2.23 | -0.86 | 1.94 | -10.64 | A_CYS42,A_HIS57,A_CYS58,A_GLN192,A_GLY193,  A_SER195. |
| 3,8-Dihydroxy-2-methylchromone |  | 3.99 | -1.48 | 2.00 | -11.17 | A_SER190,A_CYS191,A_SER195,A_GLY216, A_CYS220. |
|  |  |  |  |  |  |  |
| Paeoniflorin | MAPK10 | 6.56 | -1.46 | 4.89 | -20.44 | A_PHE58,A_VAL60,A_LYS62,A_ASN66,A_LEU67. |
| Oxypaeoniflora |  | 5.50 | -1.40 | 4.62 | -40.12 | A_CYS42,A_HIS57,A_LEU97B,A_SER190,A_GLN192,  A_SER214,A_TRP215,A_GLY216,A_GLY226. |
| Paeonol |  | 5.09 | -0.44 | 1.01 | -10.70 | A_VAL51,A_PHE58,A_ASN66,A_LEU67. |
| Quercetin |  | 5.17 | -0.56 | 5.93 | -32.53 | A_VAL51,A_VAL60,A_LYS62,A_TYR64,A_GLN65,  A_ASN66,A_LEU67. |
| Gallic acid |  | 4.00 | -0.35 | 3.85 | 9.45 | A_SER50,A_VAL51,A_VAL60,A_ASN66 |
| 3,8-Dihydroxy-2-methylchromone |  | 3.46 | -0.50 | 2.71 | -10.45 | A_VAL60,A_LYS62,A_TYR64. |
|  |  |  |  |  |  |  |
| Paeoniflorin | AKT1 | 5.71 | -1.68 | 4.31 | -85.40 | A_GLN79,A_THR82,A_ARG273,A_ASP274,  A_ASP292,A_GLY294. |
| Oxypaeoniflora |  | 5.78 | -2.38 | 5.28 | -8.89 | A_TRP80,A_SER205,A_TYR272,A_ASP274,  A_THR291,A_ASP292,A_GLY294. |
| Paeonol |  | 5.45 | -0.60 | 1.98 | -12.81 | A_GLU17,A_LYS20,A_GLU85,A_CYS310. |
| Quercetin |  | 6.49 | -0.93 | 5.35 | -12.55 | A_GLU17,A_GLU85,A_VAL271,A_TYR272. |
| Gallic acid |  | 3.28 | -0.21 | 3.83 | -54.95 | A_GLU17,A_ASN54,A_GLN79,A_ARG86,A_TYR326 |
| 3,8-Dihydroxy-2-methylchromone |  | 4.87 | -0.33 | 2.28 | -5.84 | A_TYR326. |
|  |  |  |  |  |  |  |
| Paeoniflorin | NOS3 | 5.03 | -1.14 | 4.24 | 14.50 | A_ASP1,A_GLN27,A_HIS98,A_VAL99. |
| Oxypaeoniflora |  | 7.55 | -1.01 | 4.82 | -19.29 | A_ASP1,A_GLN27,A_VAL99. |
| Paeonol |  | 3.08 | -0.66 | 1.38 | -18.69 | A_ASP1,A_VAL2,A_MET4, A_PRO101. |
| Quercetin |  | 3.08 | -0.90 | 4.63 | -26.76 | A_ASP1,A_PRO101,A_THR102,A_PHE103,A_GLY105. |
| Gallic acid |  | 2.65 | -0.91 | 4.45 | -29.34 | A_ASP1,A_MET4,A_PRO101. |
| 3,8-Dihydroxy-2-methylchromone |  | 3.69 | -0.74 | 2.51 | -11.52 | A_VAL2, A_VAL3, A_MET4, A_THR102 |

**Table S7** Binding sites of the compounds and the interaction

| Compounds | Hydrogen bond amino acids  F2(3tu7) | Hydrogen bond amino acids  MAPK14(ID =1a9u) |
| --- | --- | --- |
| paeonol | --- | ASP168, MET109 |
| quercetin | PHE227, GLU192,SER195 | ASP168,LEU104 |
| Paeoniflorin | GLY216,GLY219,CYS220,GLU192 | PHE169, LYS53 |
| oxypaeoniflorin | --- | PHE169,LYS53,ALA51,LEU104 |

**Figure S1. Inhibitory effect of 4 compounds on HUVEC(MTT)**


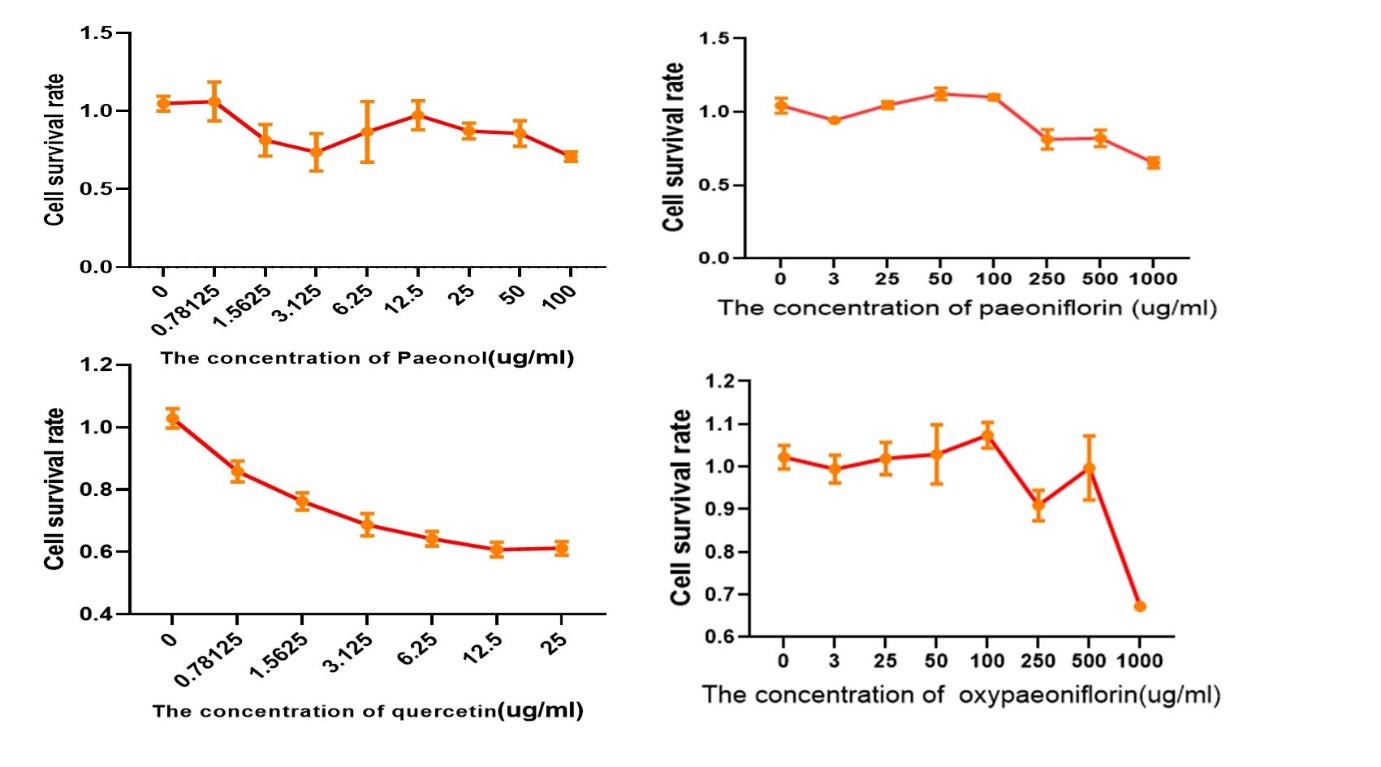

Supplement: Supplementary file 1 — Additional file 1: Table S1. The primers used in RT-qPCR . Table S2. Calibration curves, linear range of nine compounds. Table S3. Intra-day and inter-day accuracy and precision of nine compounds in rat serum. Table S4. Stability of nine compounds in rat serum (mean ± SD，n=5). Table S5. Extraction recovery and Matrix effect of nine compounds in rat serum (mean ± SD，n=5). Table S6. The molecular docking information results. Table S7. Binding sites of the compounds and the interaction. Figure S1. Inhibitory effect of 4 compounds on HUVEC(MTT). [file 13020_2022_657_MOESM1_ESM.docx]
